# Heme oxygenase metabolites improve astrocytic mitochondrial function via a Ca^2+^-dependent HIF-1α/ERRα circuit

**DOI:** 10.1371/journal.pone.0202039

**Published:** 2018-08-28

**Authors:** Yoon Kyung Choi, Joon Ha Park, Jung-A Yun, Jong-Ho Cha, Yonghee Kim, Moo-Ho Won, Kyu-Won Kim, Kwon-Soo Ha, Young-Guen Kwon, Young-Myeong Kim

**Affiliations:** 1 Department of Integrative Bioscience and Biotechnology, Konkuk University, Seoul, Republic of Korea; 2 Department of Neurobiology, School of Medicine, Kangwon National University, Chuncheon, Kangwon-do, Republic of Korea; 3 Department of Molecular and Cellular Biochemistry, School of Medicine, Kangwon National University, Chuncheon, Kangwon-do, Republic of Korea; 4 College of Pharmacy and Research Institute of Pharmaceutical Sciences, Seoul National University, Seoul, Republic of Korea; 5 Department of Biochemistry, College of Life Science and Biotechnology, Yonsei University, Seoul, Republic of Korea; University of Kansas, UNITED STATES

## Abstract

Heme oxygenase-1 (HO-1) exerts beneficial effects, including angiogenesis and energy metabolism via the hypoxia-inducible factor-1α (HIF-1α) and peroxisome-proliferator-activating receptor-γ coactivator-1α (PGC-1α)/estrogen-related receptor α (ERRα) pathways, respectively, in astrocytes. However, evidence of cross-talk between both pathways in HO metabolite-mediated mitochondrial biogenesis has not been well elucidated. Here, we found that HIF-1α was upregulated in astrocytes after ischemic brain injury following exposure to the carbon monoxide (CO)-releasing compound CORM-2. Experiments with pharmacological inhibitors and target-specific siRNAs revealed that HIF-1α levels were highly correlated with increased PGC-1α and ERRα levels, which were linked to the HO metabolites CO- and bilirubin-induced activation of apical L-type Ca^2+^ channel and sequential Ca^2+^-dependent signal transduction. Moreover, HIF-1α was stabilized in a proline hydroxylase-dependent manner by transient induction of intracellular hypoxia via the PGC-1α/ERRα-induced increases in mitochondrial biogenesis and oxygen consumption. HIF-1α knockdown blocked HO-1 system-mediated transcriptional expression of ERRα, but not of PGC-1α, suggesting a possible involvement of HIF-1α in ERRα-mediated mitochondrial biogenesis. These data suggest that the HO-1-derived metabolites, CO and bilirubin, elevate astrocytic mitochondrial function via a HIF-1α/ERRα circuit coupled with L-type Ca^2+^ channel activation and PGC-1α-mediated oxygen consumption. This circuit may play an important role in repairing neurovascular function after focal ischemic brain injury by stimulating mitochondrial biogenesis.

## Introduction

Astrocytes in the central nervous system play a key role in angiogenesis [1] and energy-metabolic activity in some pathological conditions, including cerebral ischemia [2]. Recent studies demonstrated that astrocytic function can be regulated by carbon monoxide (CO), which is endogenously produced by the catalytic action of heme oxygenase (HO). HO-1 is induced in astrocytes [3, 4], and the HO-1/CO pathway fulfills various important roles in angiogenesis and mitochondria biogenesis in astrocytes following cerebral ischemia in mice [4, 5]. The astrocytic functions are also largely associated with upregulation of peroxisome proliferators-activated receptor γ-coactivator-1α (PGC-1α), estrogen-related receptor α (ERRα), hypoxia-inducible factor-1α (HIF-1α), and vascular endothelial growth factor (VEGF).

Treatment of human astrocytes with CO-releasing molecule-2 (CORM-2) for 8 h elevates HIF-1α protein levels by promoting translational efficiency through the PI3K/Akt and MEK/ERK pathways or by increasing HO-1 protein stability by interacting with heat shock protein 90α (HSP90α) [6]. In addition, our previous study indicated that PGC-1α and ERRα proteins were detected in the recovery phase of astrocytes exposed to CORM-2 [4]. PGC-1α and ERRα have been demonstrated to play pivotal roles in HO-1/CO-mediated mitochondria biogenesis and angiogenesis in astrocytes by upregulating cytochrome *c* and VEGF expression, respectively [4, 5].

The role of signaling communication among PGC-1α, HIF-1α, and ERRα in HO-1 metabolite-induced astrocytic mitochondrial biogenesis and oxygen consumption has not been reported. Here, we dissected the signaling cascade among PGC-1α, HIF-1α, and ERRα in HO-1-mediated mitochondrial biogenesis in astrocytes. HO-1 metabolites, including CO and bilirubin, increased HIF-1α stability by sequential activation of the L-type Ca^2+^ channel, Ca^2+^/calmodulin-dependent protein kinase kinase β (CaMKKβ), and AMP-activated protein kinase α (AMPKα), resulting in increases in mitochondrial oxygen consumption (temporal hypoxia) and proline hydroxylase 2 (PHD2) inactivation. The stabilized HIF-1α further increases ERRα expression at the transcriptional level, implying a positive circuit among HIF-1α, ERRα, and mitochondrial biogenesis. Thus, the HO-1-CO/bilirubin pathway may contribute to both angiogenesis and energy metabolism, leading to potential improvement of neurovascular function after ischemic or hypoxic injury.

## Materials and methods

### Materials

Cell culture media and supplements were purchased from Invitrogen Life Technologies (Carlsbad, USA). Fetal bovine serum (FBS) was obtained from HyClone Laboratories (Logan, UT). MG132 and proteinase inhibitor cocktail were purchased from Enzo Life Sciences (Ann Arbor, USA). CORM-2 (a CO-releasing compound, [Ru(CO)_3_Cl_2_]_2_), RuCl_3_, RuCl_2_(DMSO)_4_, nifedipine, ethylene glycol-bis(2-aminoethylether)-*N*,*N*,*N′*,*N′*-tetra-acetic acid (EGTA), antimycin A, cycloheximide, hemin, and bilirubin were purchased from Sigma (St. Louis, USA). Sn(IV) protoporphyrin IX dichloride (SnPP) was purchased from Frontier Scientific (Logan, UT). ω-Conotoxin GVIA, ω-Agatoxin TK, and SNX 482 were purchased from Tocris bioscience (Bristol, BS110QL). The 4′,6-Diamidino-2-phenylindole (DAPI) was obtained from Thermo Fisher Scientific (Waltham, USA). Fluo-4 AM was purchased from Thermo Fisher Scientific (Waltham, USA). Compound C (dorsomorphin), an AMPK inhibitor, was purchased from Enzo Life Sciences (Farmingdale, NY, USA).

### Cell culture, transfection, immunofluorescence, and MitoTracker staining

Primary human brain astrocytes were purchased from the Applied Cell Biology Research Institute (Kirkland, USA). Astrocytes were cultured in Dulbecco’s modified eagle medium (DMEM) supplemented with 10% FBS and used in passages 5–9. When astrocytes reached 80% density with 10% FBS-containing DMEM, the media were replaced with serum-free DMEM. Cells were treated with RuCl_3_ (200 μM) or CORM-2 (100 μM) for 8 h in serum-free DMEM medium, washed with fresh medium, and further recovered for 24 h in serum-free DMEM medium (known as Ru/R or CO/R, respectively). RuCl_3_ was used as a control because this compound does not significantly induce HIF-1α, as occurs with RuCl_2_(DMSO)_4_ (S1 Fig). Our previous study showed that treatment of astrocytes with CORM-2 increased HIF-1α protein levels in a concentration-dependent manner [6]; thus, CORM-2 effectively increased HIF-1α level at a concentration of 100 μM, compared with that using 10 and 50 μM CORM-2. Astrocytes were grown to 70% confluence and transiently transfected with pcDNA3.1/SIRT1 (provided by Dr. Sungwoo Ryoo, Kangwon National University), pcDNA3.1/HO-1 (provided by Dr. Jozef Dulak, Jagiellonian University), or various siRNAs (50 nM) using Lipofectamine and Plus reagent (Thermo Fisher Scientific). After recovery for 12 h, cells were treated with RuCl_3_ (200 μM) or CORM-2 (100 μM) for 8 h, followed by recovery for 24 h. The siRNAs targeting human PHD2 and HIF-1α were purchased from Dharmacon (Lafayette, USA). Other commercial human siRNAs for ERRα, HO-1, LKB1, CaMKKβ, AMPKα, and PGC-1α were purchased from Santa Cruz Biotechnology (Dallas, USA). The control GFP siRNA (siGFP, 5′-GGCUACGUCCAGGAGCGCA-3′) was designed by Bioneer (Daejeon, South Korea). Astrocytes were fixed in 3.7% formaldehyde for 30 min at 23±2°C, washed gently, blocked, and incubated with the ERRα primary antibody (Santa Cruz Biotechnology) overnight at 4°C, followed by incubation with an Alexa Fluor antibody (Thermo Fisher Scientific). Nuclei were stained using DAPI. Images were obtained with a confocal microscope (Olympus FV1000). Intracellular active mitochondria levels were measured by quantitative fluorescence imaging using the mitochondria-sensitive dye MitoTracker-Red (Thermo Fisher Scientific). Astrocytes plated on 25 mm round coverslips in 6-well plates were cultured until 80% confluent. Cells were subjected to Ru/R or CO/R for 23 h with or without HIF-1α siRNA transfection. Cells were treated with 10 μM Compound C for 30 min, and 0.5 μM MitoTracker-Red was added for an additional 30 min. After washing with PBS, fluorescent images of live cells were obtained using a confocal microscope (Olympus FV1000) and were analyzed using Image J (http://rsb.info.nih.gov/ij/). The average intensity of 5 randomized cells from each image was determined.

### Measurement of intracellular Ca^2+^ concentration ([Ca^2+^])

[Ca^2+^]_i_ levels were measured by quantitative fluorescence imaging using the Ca^2+^-sensitive dye, Fluo-4. Astrocytes plated on 25 mm round coverslips in 6-well plates were cultured until 80% confluent. Cells plated on coverslips were incubated with 1 μM of the Fluo-4 for the last 30 min of 100 μM CORM-2 exposure (8 h). Subsequently, cover slips were washed with PBS, and viewed under confocal microscope (Olympus FV1000).

### Oxygen consumption analysis

Astrocytes were subjected to Ru/R or CO/R for 20 h and further incubated with 0.5 μM antimycin A, 10 μM Nifedipine, 10 μM Compound C, or 2 mM EGTA for 4 h. Real time oxygen consumption in astrocytes was measured by the Oxygen Consumption Rate Assay Kit (Cayman, Ann Arbor, USA). Astrocytes (70–80% confluent cells/150 μl/well) were prepared in non-coated regular 96 wells, and O_2_ sensor probe (10 μl) was added into each well. After covering with 100 μl of Mineral Oil, the plates were read with a filter combination of 380 nm for excitation and 650 nm for emission at 37°C (BioTek, Winooski, USA).

### Western blot analysis

Tissue samples and cells were lysed in Protein Extraction Solution (RIPA or whole cell lysate buffer). We purchased RIPA buffer from Elpis-Biotech (South Korea). Whole cell lysate buffer: 10 mM HEPES (pH 7.9), 400 mM NaCl, 0.1 mM EDTA, 5% glycerol, 1 mM DTT, and 1 mM PMSF with proteinase inhibitor cocktail (Thermo Fisher Scientific). Samples were heated with equal volumes of SDS buffer and 2-mercaptoethanol at 100°C for 5 min, and each sample was loaded onto Tris-glycine gel. After electrophoresis and transfer, PVDF membranes (Millipore) were blocked in Tris-buffered saline containing 0.1% Tween 20 and 5% skim milk (Lab Scientific). The antibodies used in this study were as follows: HIF-1α (BD Biosciences); HO-1 (Enzo Life Biosciences); LKB1, COX2, CaMKKβ, ERRα, PGC-1α, SIRT1 (Santa Cruz Biotechnology); AMPKα, p-AMPKα (Thr^172^) (Cell signaling technology); PHD2 (Novus Biologicals, Littleton, USA); β-Actin (Sigma). Membranes were incubated with peroxidase-conjugated secondary antibodies and visualized using enhanced chemiluminescence (Elpis-Biotech). Bands from western blotting (S1 File) were analyzed using Image J.

### Reverse transcriptase-polymerase chain reaction (RT-PCR)

Total RNAs were isolated from the indicated cells using Trizol reagent (Thermo Fisher Scientific). RT-PCR analysis was performed as described previously [6]. The following sets of primers were used: human HIF-1α; 5’-AGTCGGACAGCCTCAC-3’ (forward) and 5’-TGCTGCCTTGTATAGGA-3’ (reverse), human ERRα: 5’-TGAGAAGCTCTATGCCATGCCTGA-3’ (forward) and 5’-ATAGAAATGGGCCAGCACTTTGCC-3’ (reverse), and human GAPDH: 5’- CAGGGCTGCTTTTAACTCTG-3’ (forward) and 5’-TAGAGGCAGGGATGATGTTC-3’ (reverse), PCR products were analyzed on 1.2% agarose gels, and the gels were digitally imaged (BioImaging System).

### Chromatin immunoprecipitation (ChIP) assay

A chromatin immunoprecipitation (ChIP) assay was performed according to the protocol supplied by Millipore. Astrocytes were treated with Ru/R or CO/R and cross-linked with 1% formaldehyde for 10 min at 23±2°C. After sonication, chromatin was immunoprecipitated overnight with 10 μl of antibody against HIF-1α (Novus Biologicals). Targeted promoter sequences of ERRα were identified using PCR (32 cycles at 94°C for 30 s, 55°C for 30 s, and 72°C for 30 s) using primers spanning ERRα-specific promoter regions containing the binding sequence (+539 to +542, 5’-CGTG-3’) of the HIF-1α/HIF-1β complex. The primer sequences were as follows: 5′-GGAGGGCTCTATGTCTGGGA-3′ (forward) and 5′- GTAAGTGGGGAGAGCCAAGG-3′ (reverse). The products (139 bp) were identified on a 1% agarose gel.

### Focal cerebral ischemia and tissue immunohistochemistry

12 week wild-type (WT) and HO-1^+/-^ mice (BALB/c male mice, Jackson’s Laboratory) were kept in standard conditions with water and food available *ad libitum*. All experimental procedures were carried out under a protocol approved by Kangwon National University’s Animal Care and Use Committee and were in accordance with the National Institutes of Health guidelines for the care and use of laboratory animals. Focal cerebral ischemia was achieved by right carotid sheath endovascular middle cerebral artery occlusion for 2 h, according to the same protocol as previously described [4]. To confirm proper middle cerebral artery occlusion, a laser-Doppler probe (Transonic Systems Inc., USA) was fixed on the skull (2 mm posterior to the bregma and 6 mm from the midline on the right side) to measure local cortical blood flow in an area supplied by the middle cerebral artery during the operation. Only mice with ≥60% flow reduction during the ischemic period were included in this study in order to exclude incomplete ischemia. Successful occlusion was determined by a 60% decrease from baseline in local cortical blood flow. For histological analysis, mice were anesthetized with sodium pentobarbital (30 mg/kg, i.p.) and perfused transcardially with phosphate-buffered saline (PBS, pH 7.4) followed by 4% paraformaldehyde in PBS. The brains were removed and postfixed in the same fixative for 6 h at 4°C. After the brain was embedded in paraffin using standard techniques, tissues were sectioned into 10 μm sections, and sections were mounted on slides coated with 2% Elmer’s glue. The sections were treated with 0.3% H_2_O_2_ in PBS to block endogenous peroxidase activity for 30 min, and then incubated in 10% normal horse serum-supplemented PBS for 30 min. The sections were then incubated with a mouse anti-HO-1 (1:150, Enzo Life Sciences) or a mouse anti-HIF-1α antibody (1:150, Novus Biologicals) in PBS at 23±2°C. After washing three times for 10 min with PBS, sections were incubated sequentially in biotinylated goat anti-rabbit IgG or goat anti-mouse IgG (Vector Laboratories, Burlingame, USA) and peroxidase-conjugated streptavidin (Vector Laboratories) diluted 1:200 in the same solution as the primary antiserum. Between the incubations, the tissues were washed with PBS. The sections were visualized with 3,3-diaminobenzidine (0.5 mg/ml, DAB) in 0.1 M Tris buffer and mounted with Canada balsam (Junsei Chemical Co., Japan). Stained sections were subsequently examined with an inverted phase contrast microscope (Olympus, Japan). For double immunofluorescence staining, the sections were incubated with a rabbit anti-glial fibrillary acidic protein (GFAP) antibody (1:200, EMD millipore), or a mouse anti-HIF-1α antibody (1:150, Novus Biologicals) in PBS at 23±2°C, according to the same protocol as previously described [4]. After washing, the sections were then incubated in a mixture of both Cy3-conjugated goat anti-rabbit IgG (1:200; Jackson ImmunoResearch) and FITC-conjugated goat anti-mouse IgG (1:200; Jackson ImmunoResearch) for 2 h at 23±2°C. The immunoreactions were observed under the confocal microscope (Olympus FV1000, Japan).

### 2,3,5-Triphenyltetrazolium chloride (TTC) staining

Mice were euthanized and perfused transcardially with PBS. The brains were removed, and 1 mm coronal sections were dissected from the frontal pole using a mouse brain slicer (brain Matrix, ASI Instruments, Houston, TX). Six slices were selected according to the mouse brain atlas, including the main portion of the infarct. The slices were incubated for 30 min in 2% TTC solution (Sigma) at 37°C, and fixed by immersion in 4% paraformaldehyde solution in PBS for 6 h. The brain tissues were cryoprotected by infiltration with 30% sucrose overnight. Images of TTC-stained brain sections were obtained using a digital camera (Sony, Tokyo, Japan).

### Data analysis and statistics

All experiments were randomized and performed in a blinded manner. Quantification of the intensity of the protein band, which was obtained via western blot analysis, was analyzed using ImageJ (http://rsb.info.nih.gov/ij/) and normalized to the density of the actin. GraphPad Prism 6 was used for overall statistical analysis in this study. Multiple comparisons were evaluated using Tukey in One-way ANOVA plus Tukey’s test (mean ± SD). *P* < 0.05 was considered to be statistically significant.

## Results

### HO-1 and HIF-1α are co-expressed in the peri-infarct region of mouse ischemic brain

**Fig 1:**
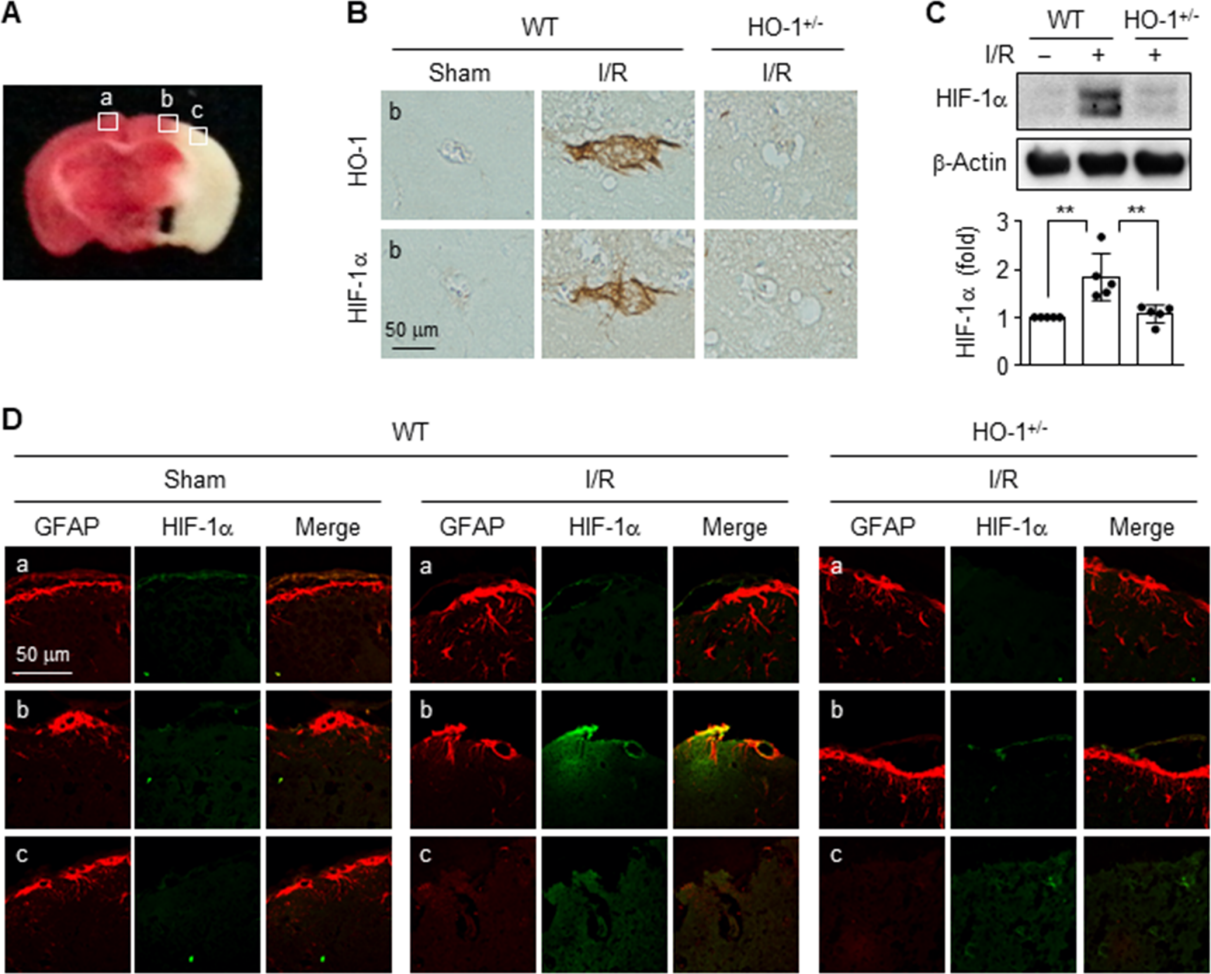
HO-1 and HIF-1α are expressed in the peri-infarct region of the ischemic mouse brain. (**A**) Representative image of the TTC stained regions (a, contralateral region; b, peri-infarct region; c, infarct region) in a mouse subjected to 2 h ischemia and 24 h reperfusion (I/R) (*n* = 3 per group). (**B**) DAB staining observed as brown color in the peri-infarct region (b) in wild-type (WT) and HO-1^*+/-*^ mice. Scale bars = 20 μm. (**C**) Expression of target proteins was determined in brain tissues using western blot analysis, and their levels were quantified (*n* = 5 per group). **P < 0.01. (**D**) WT and HO-1^*+/-*^ mice were subjected to I/R, and the brain sections (a, contralateral region; b, peri-infarct region; c, infarct region) were stained with the indicated antibodies (*n* = 4 per group). Images are representative from three individual tissues.

HIF-1α is a major transcription factor for several genes associated with angiogenesis, energy metabolism, cell survival, and neuroprotection after stroke [7, 8], and HO-1 protects against ischemia/reperfusion (I/R) after brain injury [9]. We first investigated whether HO-1 induces HIF-1α expression in the brain tissues of WT and HO^+/-^ mice following middle cerebral artery occlusion, as an experimental mouse model of cerebral I/R injury (**Fig 1A**). HO-1 expression was observed in the peri-infarct brain region of WT mice, but not in that of HO^+/-^ mice, which was similar to the expression pattern of HIF-1α (**Fig 1B**). Similar HIF-1α levels were also observed in the brain tissues of both types of mice, as assessed by western blotting (**Fig 1C**). HIF-1α immunoreactivity significantly increased only in the peri-infarct brain region of WT mice after I/R, compared to that of HO-1^*+/-*^ mice, which was localized in glial fibrillary acidic protein (GFAP)-stained astrocytes (**Fig 1D**), suggesting that HIF-1α expression is regulated by the expression status of HO-1 in ischemic astrocytes.

### Pretreated CO induces HIF-1α and ERRα expression by activating the L-type Ca^2+^ channel

**Fig 2:**
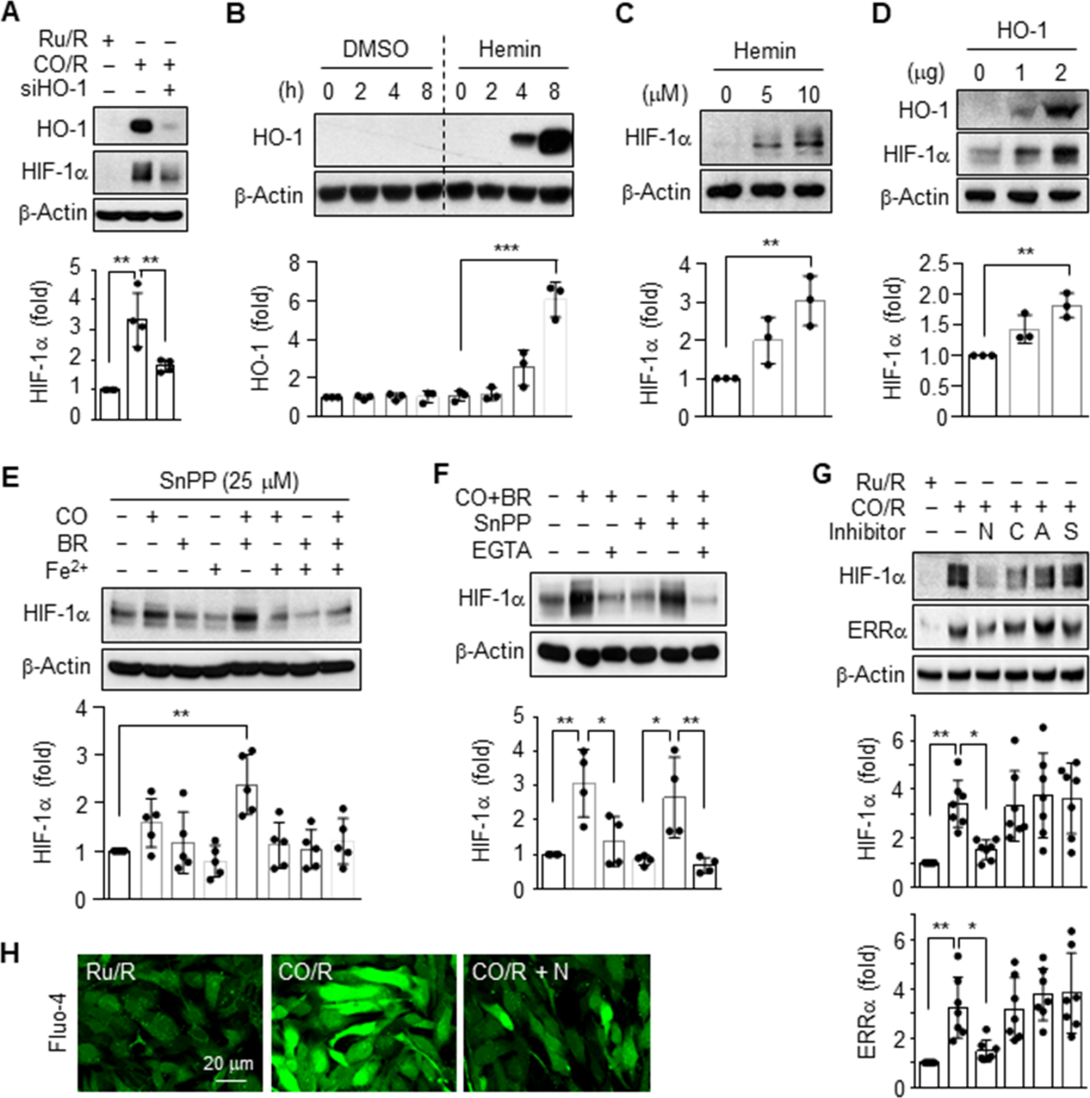
CO/R induces HIF-1α and ERRα expression via L-type Ca^2+^ channels. (**A**) Astrocytes were transfected with 50 nM control or 50 nM HO-1 siRNA and subjected to Ru/R or CO/R. Indicated protein levels were analyzed using western blotting and quantified (*n* = 4). (**B**) Astrocytes were treated with or without 10 μM Hemin for the time indicated (*n* = 3). (**C**) Astrocytes were treated with 0, 5, or 10 μM Hemin for 8 h (*n* = 3). (**D**) Cells were transfected with pcDNA3.1 mock or a pcDNA3.1/HO-1 vector and further cultured in fresh medium for 24 h (*n* = 3). (**E**) Astrocytes were pretreated with or without 25 μM SnPP, then incubated with 25 μM CORM-2 (CO), 25 μM bilirubin (BR), and 25 μM FeCl (Fe^2+^) in the presence or absence of 2 mM EGTA for 6 h. HIF-1α levels were analyzed using western blotting and quantified (*n* = 5). (**F**) Astrocytes were pretreated with or without 25 μM SnPP, then incubated with 25 μM CORM-2 (CO) and 25 μM bilirubin (BR) in the presence or absence of 2 mM EGTA for 6 h (*n* = 4). (**G**) Cells were subjected to Ru/R or CO/R, followed by treatment with 10 μM nifedipine (N), 0.3 μM ω-Conotoxin GVIA (C), 0.3 μM ω-Agatoxin TK (A), or 0.3 μM SNX 482 (S) for 4 h. HIF-1α and ERRα protein levels were quantified (*n* = 7). (**H**) [Ca^2+^]_i_ was detected using the calcium-sensitive dye Fluo-4 AM (*n* = 3). *P < 0.05; **P < 0.01.

We previously demonstrated that HO-1 expression was significantly induced by astrocytes subjected to CO/R (pretreatment with CORM-2 for 8 h and recovery for 24 h), compared to cells subjected to Ru/R (pretreatment with RuCl_3_ for 8 h and recovery for 24 h) [4]. In the present study, we examined the role of HO-1 in the energy metabolism of astrocytes exposed to CO/R or Ru/R. Astrocytes subjected to CO/R elevated the expression of HO-1 and HIF-1α, the effects of which were effectively reduced by knockdown of HO-1 using its specific siRNA (**Fig 2A**), suggesting that HIF-1α expression is regulated by HO-1 induction and activity. Treatment of human astrocytes with the HO inducer, hemin, significantly increased the HO-1 protein levels at 8 h (**Fig 2B**). A significant increase in HIF-1α protein was also observed in the cells treated with hemin (**Fig 2C**) and transfected with the HO-1 gene (**Fig 2D**). We subsequently evaluated the effects of combinations of HO byproducts (e.g. CO, bilirubin, and Fe^2+^) on HIF-1α protein levels. Since the HO-1/CO pathway promotes astrocytic function through elevation of intracellular Ca^2+^ level [Ca^2+^]_i_ [4], we examined the effects of the HO-1 inhibitor, SnPP, and the extracellular calcium chelator, EGTA, on HIF-1α expression. A slight increase in the HIF-1α level was observed in astrocytes treated with CORM-2 for 6 h, but not in those treated with bilirubin and Fe^2+^, and this increase was further significantly elevated by co-treatment of CORM-2 with bilirubin, but not co-treatment with Fe^2+^ (**Fig 2E**). Notably, the increased HIF-1α level was effectively blocked by EGTA, but not by SnPP (**Fig 2F**). This suggests that extracellular Ca^2+^ is essential for CO/bilirubin-mediated HIF-1α expression. Since the pretreated CO-mediated influx of extracellular Ca^2+^ is largely promoted by activation of voltage-dependent Ca^2+^ channels [5], we examined which type of Ca^2+^ channel is involved in CO/R-mediated expression of HIF-1α using several Ca^2+^ channel inhibitors. Among the different types of voltage-dependent Ca^2+^ channel blockers, the L-type Ca^2+^-channel blocker, nifedipine, but not other inhibitors of N-type (ω-Conotoxin GVIA), P/Q-type (ω-Agatoxin TK), or R-type channels (SNX 482), suppressed the CO/R-evoked increase in HIF-1α (**Fig 2G**). This suppressive effect was highly correlated with the protein level of ERRα (a PGC-1α-coupled transcription factor), as an important player in neuroprotection [10] (**Fig 2G**). As expected, a CO/R-mediated increase in intracellular Ca^2+^ level was blocked by nifedipine (**Fig 2H**). Collectively, these data suggest that the HO-1 metabolites, CO and bilirubin, stimulate expression of HIF-1α and ERRα through influx of extracellular Ca^2+^ by activating L-type Ca^2+^ channels.

### CO/R induces HIF-1α stability via the activation of CaMKKβ/AMPKα axis

**Fig 3:**
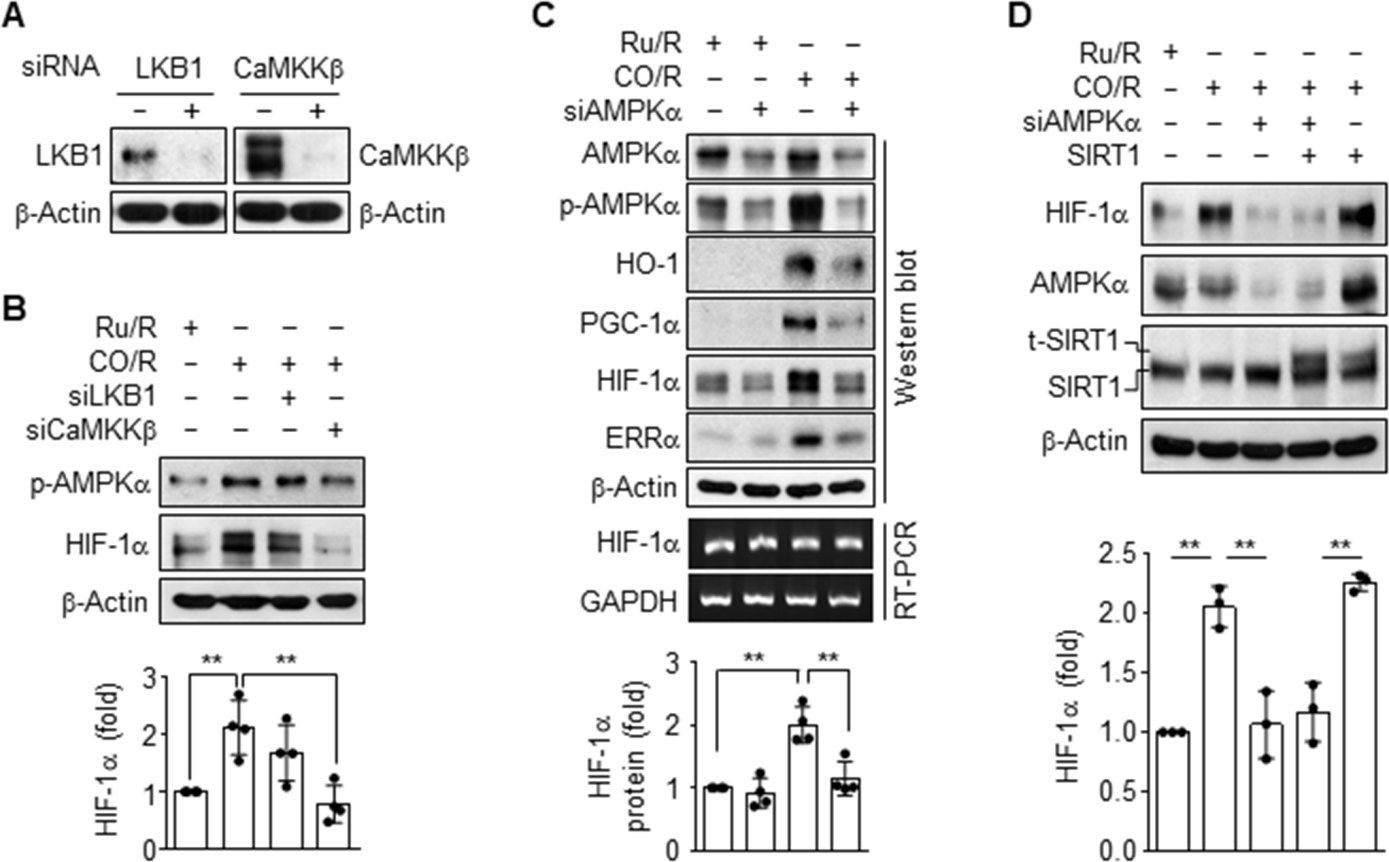
CO/R induces HIF-1α stability via the Ca^2+^-mediated CaMKKβ/AMPKα pathway. (**A-C**) Astrocytes were transfected with control or indicated siRNAs and subjected to Ru/R or CO/R. Target protein levels in whole cell lysate were detected via western blotting (*n* = 4). Target mRNA levels were detected by RT-PCR. (**D**) Cells were transfected with 50 nM control or AMPKα siRNA alone or in combination with 2 μg SIRT1 plasmid and subjected to Ru/R or CO/R. Protein levels were determined via western blotting (*n* = 3). t-SIRT1 indicates transfected SIRT1 protein, and HIF-1α protein levels were quantified (*n* = 3). **P < 0.01.

In addition to elevation of HIF-1α stability and ERRα activity, CO/R can stimulate Ca^2+^-dependent activation of the energy-sensing kinase AMPK [4]. Therefore, we examined the relationship or hierarchy among these signaling events in astrocytic HIF-1α expression in the CO/R condition. The CO/R-induced increases in AMPKα phosphorylation and HIF-1α level were remarkably reduced by knockdown of CaMKKβ, but not by knockdown of LKB1, two upstream signal mediators of AMPK (**Fig 3A and 3B**). Notably, AMPKα knockdown blocked the CO/R-mediated increases in HIF-1α protein and in HO-1, PGC-1α, and ERRα expression, without affecting HIF-1α mRNA level (**Fig 3C**). We further investigated the possibility that post-translational modification of HIF-1α by sirtuin 1 (SIRT1, NAD-dependent deacetylase) regulates the HIF-1α protein stability. However, overexpression of SIRT1 did not alter the CO/R-mediated increase in HIF-1α level or the inhibitory effect of AMPKα knockdown on CO/R-mediated elevation of HIF-1α levels (**Fig 3D**). These data collectively suggest that CO/R increases HIF-1α protein levels at the post-translational level via the Ca^2+^-dependent CaMKKβ/AMPKα axis, but not via SIRT1 activity.

### CO/R increases HIF-1α stability via increased mitochondrial oxygen consumption

**Fig 4:**
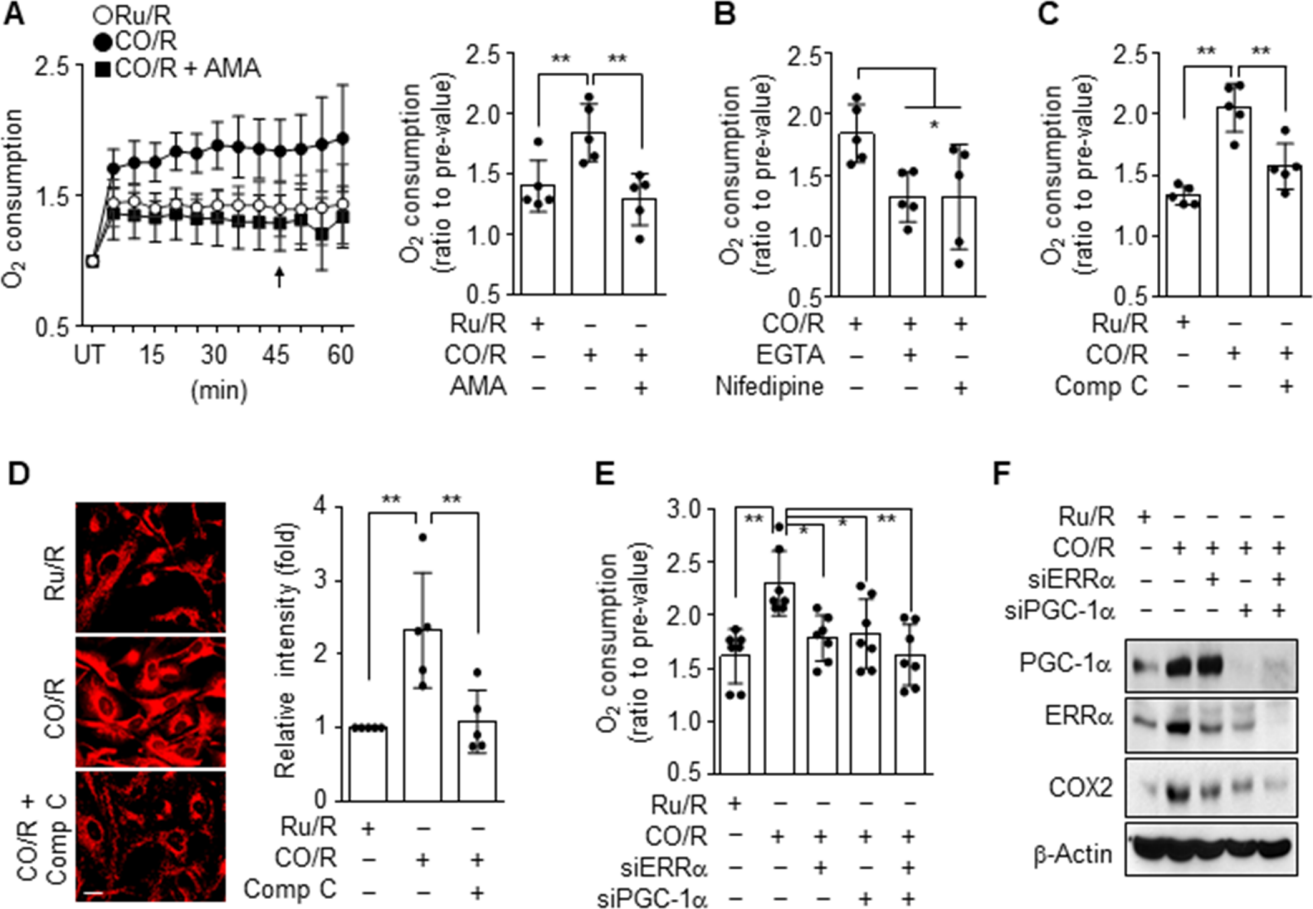
CO/R enhances oxygen consumption. (**A-D**) Astrocytes were subjected to Ru/R or CO/R and further incubated with or without 0.5 μM Antimycin A (AMA), 2 mM EGTA, 10 μM Nifedipine, or 10 μM Compound C (Comp C) for 4 h. UT indicates untreated control cells. (**A-B**) Mitochondria oxygen consumption was detected by the Oxygen Consumption Rate Assay Kit, and the quantified graph was generated at 45 min (*n* = 5). (**C**) Mitochondrial oxygen consumption was detected by the Oxygen Consumption Rate Assay Kit (*n* = 5). (**D**) Mitochondrial biogenesis was determined by staining with MitoTracker (*n* = 5). (**E-F**) Astrocytes were transfected with 50 nM control, ERRα or PGC-1α siRNA, and subjected to Ru/R or CO/R. (**E**) Oxygen consumption was measured and quantified at 45 min (*n* = 7). (**F**) Indicated proteins were detected via western blotting. *P < 0.05; **P < 0.01.

HIF-1α level is largely dependent on intracellular oxygen tension [11], which is regulated by the rate of mitochondrial oxygen consumption [12]. We examined whether CO/R increases mitochondrial oxygen consumption, as a representative index of transient intracellular low oxygen tension or hypoxic state, which is essential for HIF-1α stabilization [13]. Astrocytes subjected to CO/R significantly increased oxygen consumption, and this increase was decreased to a level similar to that of the control by treatment with antimycin A, an inhibitor of complex III in the mitochondrial electron transport chain (**Fig 4A**). The CO/R-induced increase in oxygen consumption was also blocked by treatment with EGTA, Nifedipine, and the AMPK inhibitor, Compound C (**Fig 4B and 4C**). To examine how CO/R increases mitochondrial oxygen consumption, we determined the mitochondrial contents by staining with MitoTracker. As expected, astrocytes subjected to CO/R elevated mitochondrial contents, and this increase was reduced by treatment with Compound C (**Fig 4D**), indicating that the CO/R-induced increase in astrocytic oxygen consumption is associated with increased mitochondrial contents. Although AMPK does not directly induce mitochondrial biogenesis, it can stimulate mitochondrial biogenesis via activation of PGC-1α and upregulation of ERRα [4]. We next examined the effects of PGC-1α and ERRα on mitochondrial oxygen consumption in response to CO/R. The CO/R-induced increase in astrocytic oxygen consumption was reduced by knockdown of PGC-1α or ERRα and further decreased by combined knockdown of both genes (**Fig 4E**). In a similar manner, the suppressive effects of PGC-1α or ERRα knockdown on mitochondrial cytochrome c oxidase II (COX2) expression were observed in astrocytes subjected to CO/R (**Fig 4F**). These results suggest that CO/R induces transient intracellular hypoxia by enhancing oxygen consumption, due to increased mitochondrial biogenesis in an L-type Ca^2+^ channel-dependent signaling manner.

### CO/R promotes HIF-1α stabilization in a PHD-dependent manner

**Fig 5:**
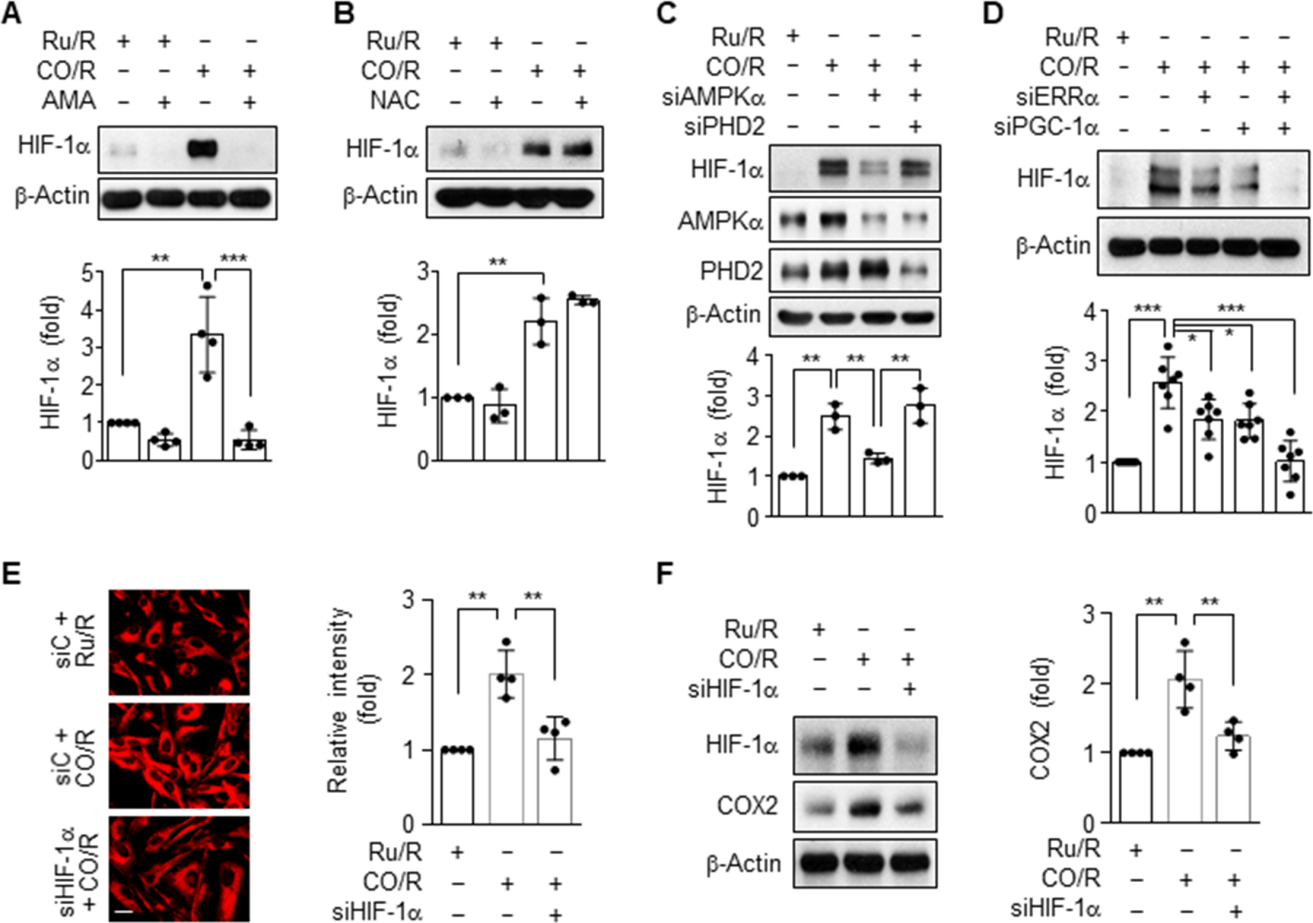
CO/R regulates HIF-1α stability via the PHD2-dependent pathway. (**A-B**) Astrocytes were subjected to Ru/R or CO/R and further incubated with or without 0.5 μM Antimycin A (AMA) (*n* = 4), or 1 mM *N*-acetylcysteine (NAC) (*n* = 3) for 4 h. Indicated proteins were detected via western blotting. (**C**) Astrocytes were transfected with 50 nM control, AMPKα, or PHD2 siRNA and subjected to Ru/R or CO/R. Indicated proteins were detected via western blotting (*n* = 3). (**D**) Astrocytes were transfected with 50 nM control, ERRα or PGC-1α siRNA, and subjected to Ru/R or CO/R. Indicated proteins were detected via western blotting and quantified (*n* = 7). (**E**) Mitochondrial biogenesis was determined by staining with MitoTracker (*n* = 4). (**F**) Astrocytes were transfected with 50 nM of control, or HIF-1α siRNA, and subjected to Ru/R or CO/R. Indicated proteins were detected via western blotting (*n* = 4). *P < 0.05; **P < 0.01; ***P < 0.001.

Since the stability of HIF-α is increased by hypoxia and reactive oxygen species (ROS) generated during mitochondrial respiration [12, 14], we next examined the roles of oxygen consumption and ROS generation in the CO/R-induced increase in HIF-α levels. Upregulation of HIF-1α by CO/R was completely blocked by treatment with antimycin A, but not by treatment with the antioxidant, *N*-acetylcysteine (NAC) (**Fig 5A and 5B**), suggesting that the CO/R-induced HIF-1α expression is due to mitochondrial oxygen consumption, but not due to ROS generation. HIF-1α is rapidly degraded by hydroxylation at two proline residues within its degradation domain by PHDs under normal oxygen tension, whereas it is stabilized by inactivation of PHD activity under hypoxic conditions [15]. We further examined the role of PHDs in CO/R-induced stabilization of HIF-1α. PHDs have three isoforms (PHD1, PHD2, and PHD3) and exhibit some degree of specificity for different prolyl hydroxylation sites within each HIF-α subunit, with PHD2 primarily hydroxylating HIF-1α [16]. Knockdown of AMPKα decreased CO/R-induced HIF-1α expression, and this inhibitory effect was fully reversed when cells were co-transfected with PHD2 siRNA (**Fig 5C**). This suggests that CO/R induces HIF-1α stabilization via increased oxygen consumption and subsequent inhibition of PHD activity. We next examined the effects of PGC-1α and ERRα, as stimulators of mitochondrial biogenesis, on HIF-α levels. Upregulation of HIF-1α by CO/R was ameliorated by knockdown of either PGC-1α or ERRα, and synergistically blocked by combined knockdown of both genes (**Fig 5D**). To examine whether HIF-1α is involved in CO/R-mediated increases in mitochondrial oxygen consumption, we determined the mitochondrial contents by staining with MitoTracker. Notably, astrocytes subjected to CO/R showed increased mitochondrial contents, and this increase was reduced by knockdown of HIF-1α (**Fig 5E**). In addition, the suppressive effect of HIF-1α knockdown on mitochondrial cytochrome c oxidase II (COX2) expression was observed in astrocytes subjected to CO/R (**Fig 5F**). These data suggest that CO/R enhances AMPKα-PGC-1α-ERRα-mediated mitochondrial biogenesis and oxygen consumption, leading to decreased PHD activity and consequently increased HIF-1α stabilization. HIF-1α stabilization further activates mitochondrial biogenesis, indicating a potential positive feedback loop between HIF-1α and mitochondrial stimulators.

#### CO/R induces HIF-1α-dependent ERRα expression

**Fig 6:**
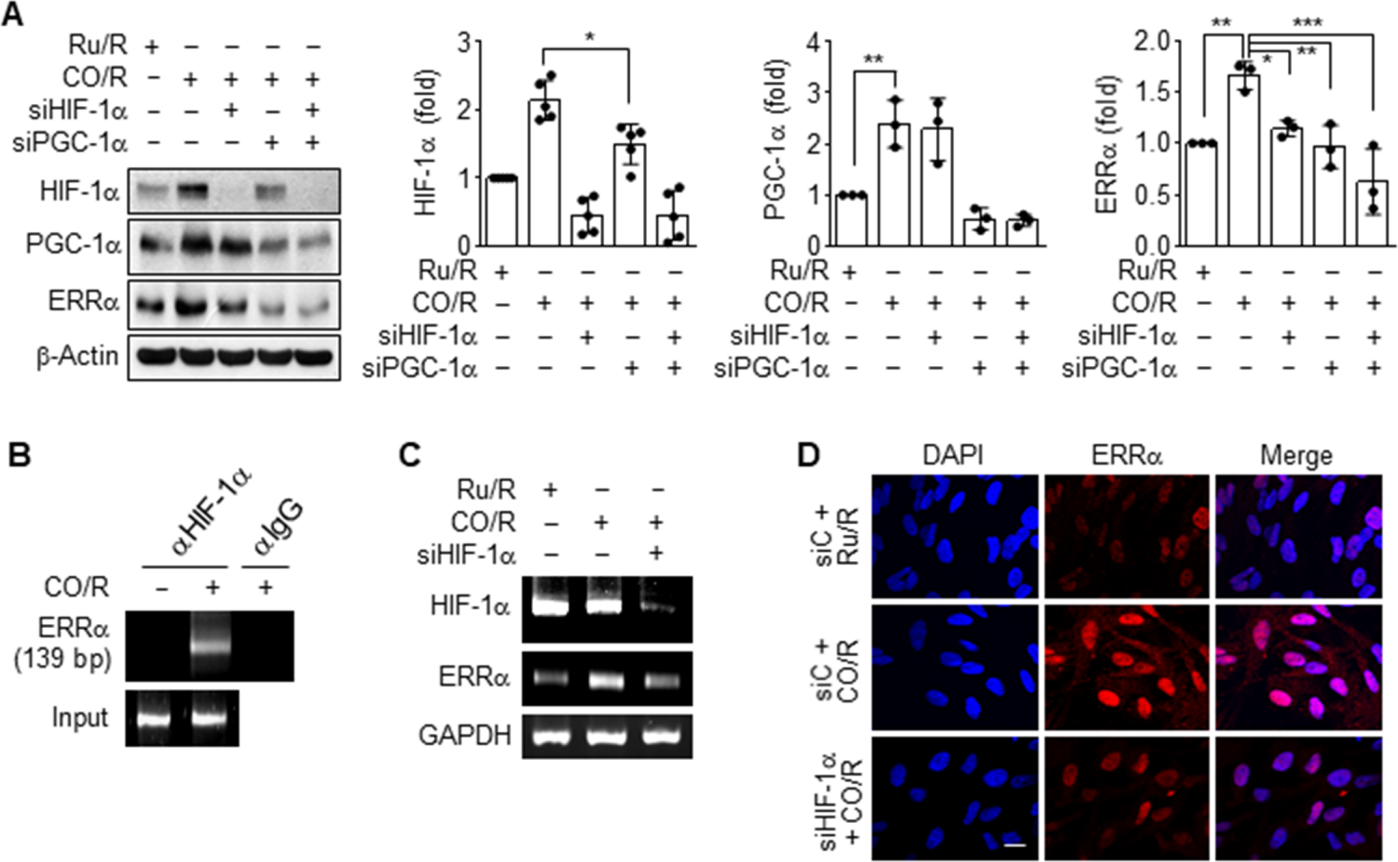
CO/R induces HIF-1α-dependent ERRα expression. (**A**) Astrocytes were transfected with control, HIF-1α or siPGC-1α siRNA, and subjected to Ru/R or CO/R. Target protein levels were detected via western blotting. The protein levels were quantified. (**B**) ChIP analysis indicated the binding ability of HIF-1α to the putative HIF-1 binding site (+539 to +542) of the ERRα promoter region of astrocytes subjected to Ru/R or CO/R. (**C-D**) Astrocytes were transfected with control or HIF-1α siRNA and subjected to Ru/R or CO/R. (**C**) Target mRNA levels were detected by RT-PCR. (**D**) ERRα expression was detected by immunocytochemistry. Nuclei were stained with DAPI. Scale bar = 10 μm.

Since HIF-1α stimulates angiogenesis and energy metabolism in pathological conditions [4, 5, 17], possibly via cross-talk with PGC-1α and ERRα [18–20], we dissected the signaling network among them in astrocytes subjected to CO/R. Knockdown of HIF-1α significantly reduced CO/R-induced expression of ERRα, but not of PGC-1α, and knockdown of PGC-1α partially inhibited HIF-1α level and markedly reduced ERRα expression (**Fig 6A**). As expected, the combined knockdown of HIF-1α and PGC-1α synergistically blocked the CO/R-induced increase in ERRα expression (**Fig 6A**). This suggests that CO/R stimulates the PGC-1α/HIF-1/ERRα circuit in astrocytes. Therefore, we assessed the interaction of HIF-1 with the ERRα promoter. A ChIP assay revealed that CO/R increased the binding ability of HIF-1α to its putative binding site within the ERRα promoter in astrocytes (**Fig 6B**). Moreover, HIF-1α knockdown markedly decreased the CO/R-induced increase in ERRα mRNA levels (**Fig 6C**). Consistent with this, ERRα protein accumulated in the nuclei of astrocytes exposed to CO/R, compared to astrocytes exposed to Ru/R, and this accumulation was abolished by transfection with HIF-1α siRNA (**Fig 6D**). These data suggest that HO-1 induction and its catalytic products, CO and bilirubin, are essential for mitochondrial biogenesis by stimulating the sequential PGC-1α/HIF-1α/ERRα pathway in addition to the HIF-1α/ERRα circuit.

## Discussion

Astrocytes play a key role in maintaining vascular function and neuroprotection in ischemic diseases [2, 21] by increasing neurogenic and angiogenic factors including VEGF [22–24] in addition to stimulating energy metabolism [25]. HIF-1α, PGC-1α, and ERRα are associated with expression of VEGF and mitochondrial genes [18, 26]; however, reciprocal cross-talk among these molecules in astrocytic mitochondrial function has not been elucidated, particularly under the condition of HO-1 induction. We here found the signaling cross-talk among these molecules promotes mitochondrial biogenesis in astrocytes subjected to CO/R that stimulate HO-1 induction. CO/R-induced HO-1 initially increased [Ca^2+^]_i_ by activating L-type voltage-gated Ca^2+^ channels, subsequently leading to CaMKKβ-mediated AMPKα activation. This signal pathway promoted the expression of HIF-1α, PGC-1α, and ERRα, which were evidently dependent of HO-1 induction in CO/R-exposed astrocytes. Our data indicated that HO-1 induction elicited the signal cross-talk and circuit of the PGC-1α/HIF-1α/ERRα axis that is essential for improvement of astrocytic energy metabolism via mitochondrial biogenesis. Considering the crucial role of astrocytes in the neurovascular unit, our data suggest that CO/R-induced HO-1 expression and its metabolites, CO and bilirubin, promote endogenous repair processes after ischemic brain injury through mitochondrial biogenesis and angiogenesis via signaling and reciprocal cross-talk among PGC-1α, HIF-1α, and ERRα.

**Fig 7:**
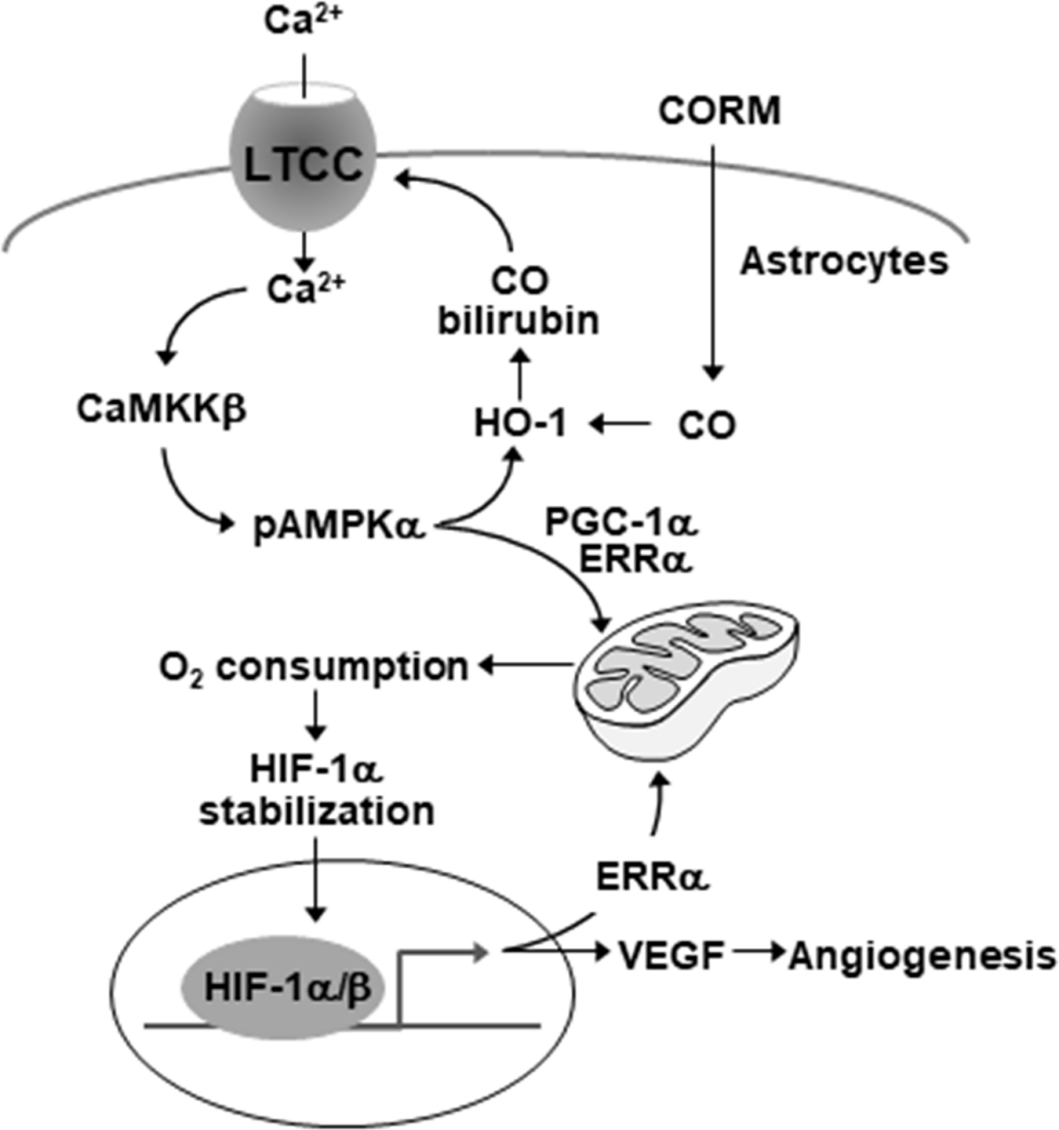
Schematic diagram indicating that the HO-1 metabolites stimulates the reciprocal circuit of HIF-1α/ERRα in mitochondrial biogenesis via the Ca^2+^-dependent signaling cascade in astrocytes. LTCC = L-type Ca^2+^ channel.

We previously demonstrated that astrocytic induction of HO-1 by CO/R plays an important role in functional improvement of the neurovascular units via endogenous production of heme catabolic products, including CO and bilirubin [4]. Bilirubin is generated from the first heme metabolite, biliverdin, by the NADH-dependent reaction of biliverdin reductase and exhibits high antioxidant and anti-inflammatory activity [27]. Bilirubin is highly lipophilic and can diffuse into the cells via the lipid bilayers [28]. CO can also freely cross the plasma membrane. Both metabolites promote angiogenesis and mitochondrial biogenesis by increasing HIF-1α, PGC-1α, and ERRα expression [4, 5]. PGC-1α and ERRα are involved in mitochondrial biogenesis [5], and HIF-1α regulates the expression of several enzymes in the glycolytic pathway instead of the tricarboxylic acid cycle, in addition to expression of the glucose transporters glucose transporter1 (GLUT1) and GLUT3 which mediate cellular glucose uptake [29, 30]. However, the role of HIF-1α in mitochondrial function and biogenesis has not been clearly elucidated. Notably, our data indicated that HIF-1α can improve oxygen-dependent energy metabolism through mitochondrial biogenesis, largely by a signaling circuit with the PGC-1α/HIF-1α/ERR axis (**Fig 7**).

PGC-1α acts as a master regulator of mitochondrial biogenesis and function via the induction and activation of several nuclear transcription factors, such as NRF-1 [31] and ERRα [32]. In addition, the transcriptional activity of ERRα is largely dependent on the presence of PGC-1α, which is expressed at low basal levels under normal conditions and is induced by energy stress [33]. Consistent with previous results [5], the present findings revealed that HO-1 metabolites enhanced both PGC-1α and ERRα expression through the Ca^2+^/CaMKK/AMPK pathway in astrocytes, resulting in an increase in mitochondrial biogenesis and respiration. This sequential event then causes transient intracellular hypoxia and subsequent stabilization of the HIF-1α protein. Notably, PGC-1α knockdown suppressed both HIF-1α and ERRα, and HIF-1α knockdown inhibited expression of ERRα, but not expression of PGC-1α. These results suggest that HIF-1α is downstream of PGC-1α and upstream of ERRα in the CO- and bilirubin-mediated signaling cascade. Similarly, it has been demonstrated that ectopic expression PGC-1α facilitated HIF-1α stabilization as a result of increased oxygen consumption after mitochondrial biogenesis, consequently leading to stimulation of HIF-1α-dependent gene expression [12].

HIF-1α not only promotes angiogenesis via VEGF expression but also stimulates anaerobic metabolism of glucose via upregulation of several glycolytic enzymes. However, HIF-1α represses mitochondrial biogenesis/function and oxygen consumption in renal carcinoma cells by inducing pyruvate dehydrogenase kinase 1 [34] or inhibiting the C-MYC/PGC-1β axis [35]. In contrast, hypoxia that stimulated HIF-1α expression significantly induced mitochondrial biogenesis in cultured cardiac myocytes and in the skeletal muscle of rats, although the underlying mechanism has not been elucidated [36, 37]. It has been demonstrated that HO-1-derived CO promotes angiogenesis and mitochondrial biogenesis via upregulation of HIF-1α and ERRα [4, 5], suggesting that reciprocal cross-talk between HIF-1α and ERRα is essential for CO-mediated cellular function. Indeed, the present results clearly demonstrated that elevated HIF-1α positively regulated ERRα expression in astrocytes subjected to CO/R at the transcriptional level, resulting in mitochondrial biogenesis. This suggests that HIF-1α is coupled to ERRα-mediated mitochondrial biogenesis. Thus, the heme degradation products, CO and bilirubin, synergistically enforce metabolic reprogramming in the process of recovery following ischemic brain injury by increasing glycolytic activity, mitochondrial biogenesis, and angiogenesis via the HIF-1α/ERRα axis.

HIF-1α level and its activity are regulated by several post-translational modifications, such as hydroxylation [15, 38] and acetylation [11, 39, 40], respectively. Our data indicated that astrocytic HO-1 induction increased HIF-1α levels in a mouse model of ischemia/reperfusion injury. The HO-1 metabolites, CO and bilirubin, enhanced HIF-1α stabilization in astrocytes by eliciting the sequential activation of the CaMKKβ/AMPKα/PGC-1α pathway. This pathway increased mitochondrial biogenesis and oxygen consumption, resulting in transient intracellular hypoxia that inhibited PHD activity and HIF-1α degradation. It has been proposed that HIF-1α can be stabilized or activated by SIRT1 [39, 40], which can be activated in astrocytes subjected to CO/R [4]. However, our data indicated that SIRT1 was not involved in CO/R-mediated HIF-1α stabilization. We propose the novel concept that HO-1 facilitates HIF-1α stabilization in a PHD-dependent manner.

PHD enzymes are iron-containing dioxygenases that use molecular oxygen and 2-oxoglutarate as co-substrates, and their activity is dependent on oxygen concentrations or ROS levels. Under normoxic conditions, PHD hydroxylates two proline residues at Pro402 and Pro564 that are recognized by the von Hippel-Lindau protein of the E3 ubiquitin ligase complex and targeted for proteasomal degradation [15, 41, 42]. On the other hand, hypoxia inhibits PHD activity, allowing for stabilization and accumulation of HIF-1α, which then associates with its dimerization partner, HIF-1β, to form the HIF-1 transcription factor [43]. In addition, PHD activity is inhibited via oxidation of Fe(II) to Fe(III) by ROS. Our data demonstrated that elevated HIF-1 levels in astrocytes subjected to CO/R were reduced by treatment with the mitochondrial complex III inhibitor, antimycin A, but not by the antioxidant, NAC. We also confirmed that CO/R treatment increased astrocytic mitochondrial biogenesis and oxygen consumption via either PGC-1α or ERRα, leading to transient induction of intracellular hypoxia that inhibited PHD activity and HIF-1α stabilization. Our proposed mechanism is novel, and differs from previous observations stating that the CO-mediated increase in HIF-1α levels is associated with promotion of protein translational efficiency or HSP90α-dependent prevention of proteasomal degradation [6]. Our findings suggest that HO-1-mediated inhibition of PHD activity is mediated by transient hypoxia via PGC-1α or ERRα-dependent mitochondrial biogenesis.

HIF-1α stimulates expression of VEGF, which is essential for neurogenesis and angiogenesis [17, 23, 44, 45]. The PGC-1α/ERRα axis also promotes VEGF expression [18] in addition to mitochondrial biogenesis [5]. These observations suggest that HIF-1α may communicate with the PGC-1α/ERRα axis. Indeed, our data revealed that HIF-1α stimulated transcriptional expression of ERRα by binding to a putative HIF-1-binding sequence (+539 to +542, 5’-CGTG-3’) within the promoter region of the ERRα gene. This reveals evidence that HIF-1α acts as a transcription factor for ERRα expression, which facilitates mitochondrial biogenesis. Therefore, there is reciprocal and dynamic coordination among PGC-1α, HIF-1α, and ERRα in astrocytic mitochondrial biogenesis following subjection to CO/R.

The HO-1-derived CO/bilirubin pathway plays an important role in improvement of neurovascular function by eliciting communication between HIF-1α and the PGC-1α/ERRα axis [4–6]. The PGC-1α/ERRα axis is triggered by extracellular Ca^2+^ influx via activation of L-type Ca^2+^ channels in CO/R-exposed astrocytes [4]. These observations suggest that HO-1/CO-mediated Ca^2+^ influx can elevate HIF-1α levels via the CaMKKβ/AMPKα-dependent PGC-1α/ERRα pathway, which elicits mitochondria oxygen consumption and transient intracellular hypoxia. Consistent with this suggestion, we found that the CO/R-induced increase in [Ca^2+^]_i_ plays an important role in HIF-1α-mediated mitochondrial biogenesis, as confirmed by the inhibitory effect of nifedipine and EGTA on the CO/R-induced increase in HIF-1α and mitochondrial respiration. These results suggest that the CO- and bilirubin-mediated Ca^2+^ influx plays a crucial role in rescuing neurovascular function after focal ischemic brain injury by promoting angiogenesis and energy metabolism via reciprocal cross-talk among PGC-1α, HIF-1α, and ERRα. Although we did not measure the levels of bilirubin and CO in ischemic brain tissues, several studies demonstrated that HO-1-deficient mice showed exaggerated cerebral damage and decreased neurogenesis after cerebral ischemia [46, 47]. These results strongly suggested that HO-1-derived metabolites play an important role in astrocytic function, which may be associated with mitochondrial biogenesis.

In conclusion, our present data demonstrate that HO-1 metabolites improve astrocytic function via mitochondrial biogenesis by triggering the novel signal circuit of a Ca^2+^-dependent HIF-1α/ERRα axis. The circuit appears to be stimulated by a sequential signal cascade of L-type Ca^2+^ channel-dependent Ca^2+^ influx, CaMKKβ-mediated AMPKα activation, PGC-1α-induced mitochondrial respiration and cellular hypoxia, HIF-1α stabilization, and ERRα upregulation (**Fig 7**). Thus, the HO-1-derived CO and bilirubin system may facilitate improved function of neurovascular units after cerebral injury by activating a reciprocal HIF-1α/ERRα axis, which stimulates mitochondrial biogenesis and angiogenesis.

## Supporting information

S1 Fig HIF-1α is expressed in CORM-2-treated astrocytes. (A) Astrocytes were exposed to 200 μM RuCl_3_ and 100 μM CORM-2 for the indicated time (pretreatment). After an 8 h treatment, cells were incubated in fresh media for 4, 16, and 24 h (recovery). HIF-1α protein levels were determined in cell lysates by Western blotting (n = 3). *P < 0.05. (B-C) Astrocytes were exposed to 200 μM RuCl_2_(DMSO)_4_, 200 μM RuCl_3_ or 100 μM CORM-2. After an 8 h treatment, cells were incubated in fresh media for 24 h (recovery). HIF-1α expression was not induced by ether reagent (RuCl_2_(DMSO)_4_ and RuCl_3_) compared with that using CORM-2.
(TIF)

S1 File Results from western blotting were shown. Membranes obtained from western blotting were demonstrated in this file.
(PDF)
