# Supplementary figures and images for: Heme oxygenase metabolites improve astrocytic mitochondrial function via a Ca2+-dependent HIF-1α/ERRα circuit
Source: PLoS One. 2018 Aug 28;13(8):e0202039. doi: 10.1371/journal.pone.0202039 (PMC6112640; doi:10.1371/journal.pone.0202039)

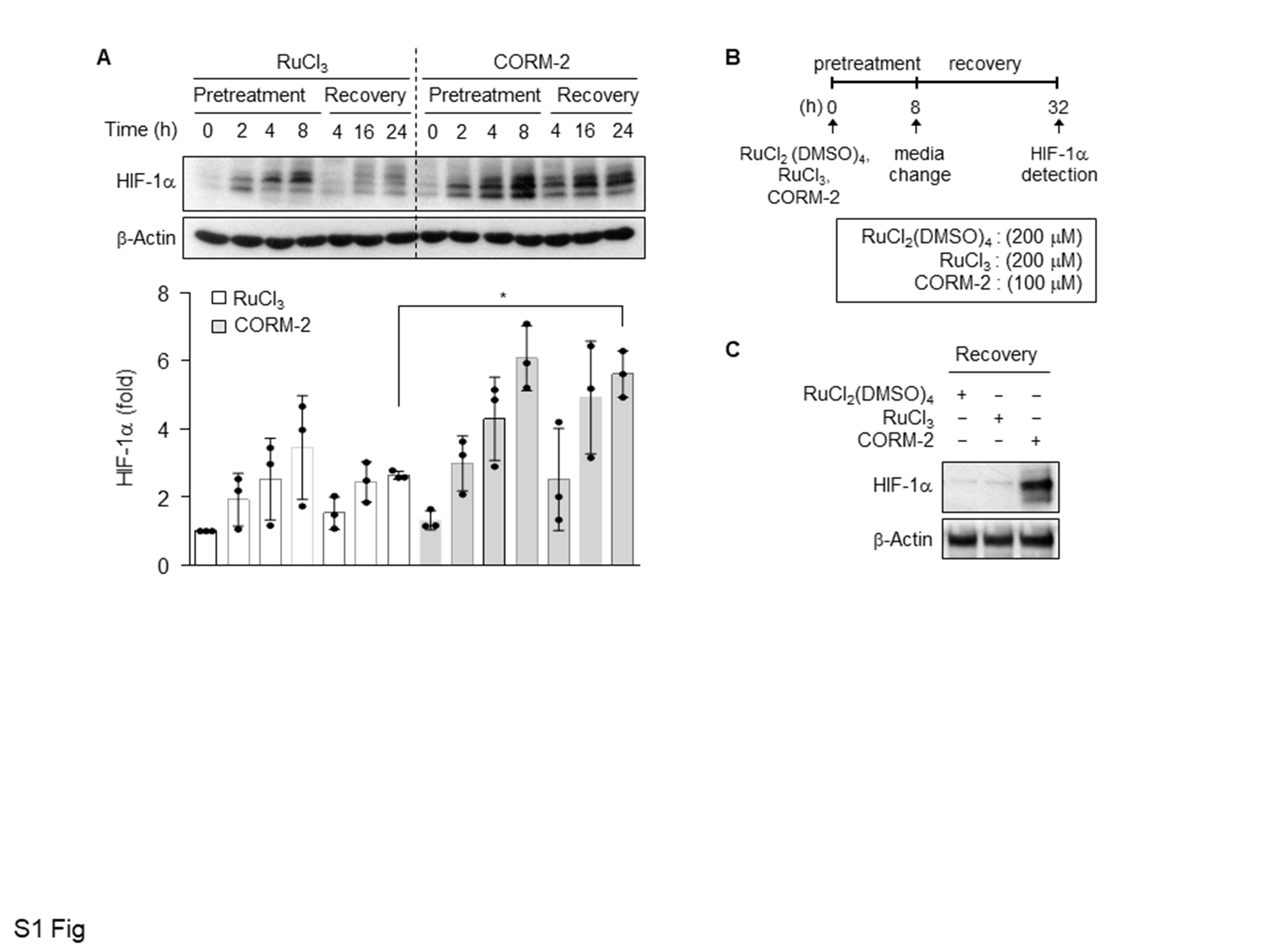

Supplement: S1 Fig — (A) Astrocytes were exposed to 200 μM RuCl3 and 100 μM CORM-2 for the indicated time (pretreatment). After an 8 h treatment, cells were incubated in fresh media for 4, 16, and 24 h (recovery). HIF-1α protein levels were determined in cell lysates by Western blotting (n = 3). *P < 0.05. (B-C) Astrocytes were exposed to 200 μM RuCl2(DMSO)4, 200 μM RuCl3 or 100 μM CORM-2. After an 8 h treatment, cells were incubated in fresh media for 24 h (recovery). HIF-1α expression was not induced by ether reagent (RuCl2(DMSO)4 and RuCl3) compared with that using CORM-2. (TIF) [file pone.0202039.s001.tif]
