# Supplementary material for: Heme oxygenase metabolites improve astrocytic mitochondrial function via a Ca2+-dependent HIF-1α/ERRα circuit
Source: PLoS One. 2018 Aug 28;13(8):e0202039. doi: 10.1371/journal.pone.0202039 (PMC6112640; doi:10.1371/journal.pone.0202039)

Fig 1C

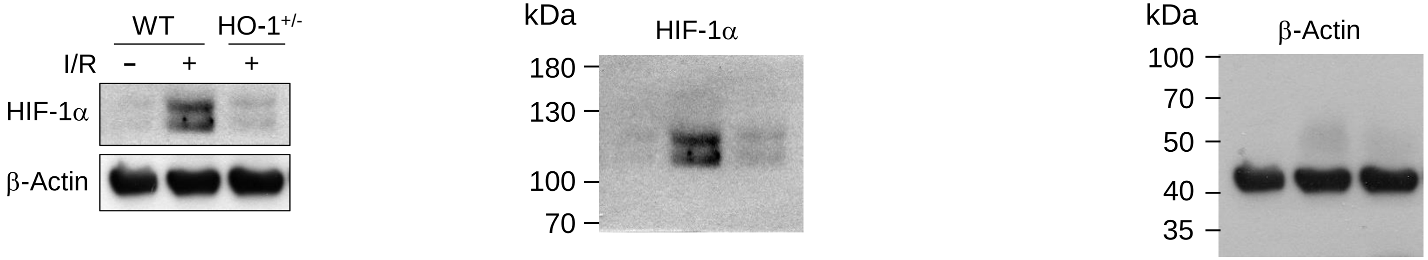

Fig 2A

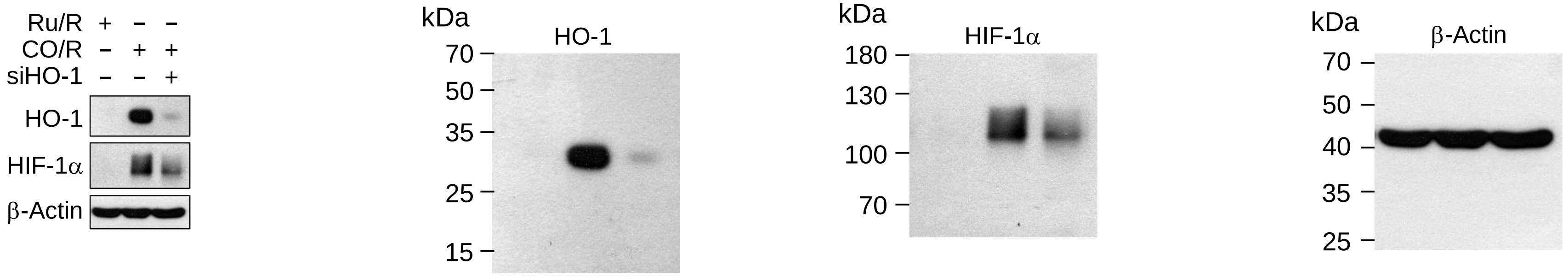

Fig 2B

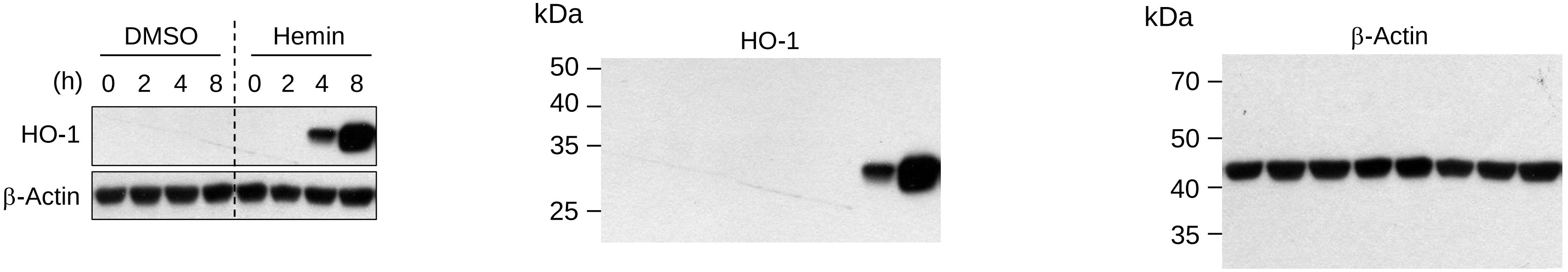

Fig 2C

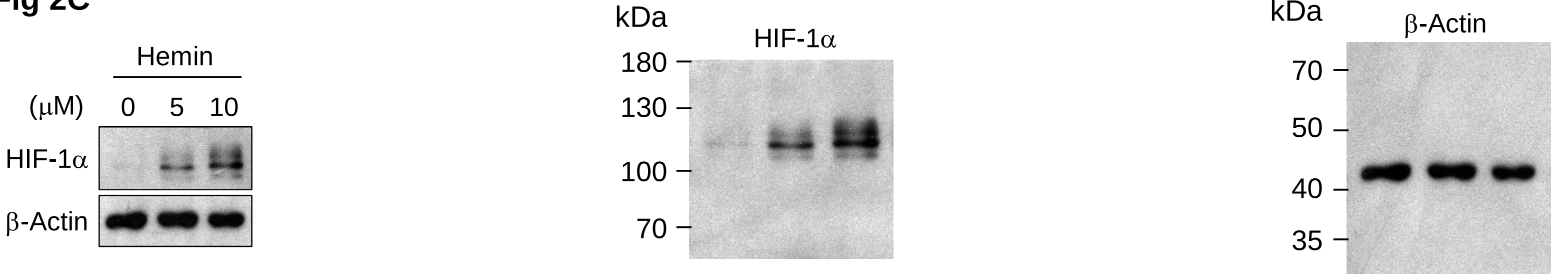

Fig 2D

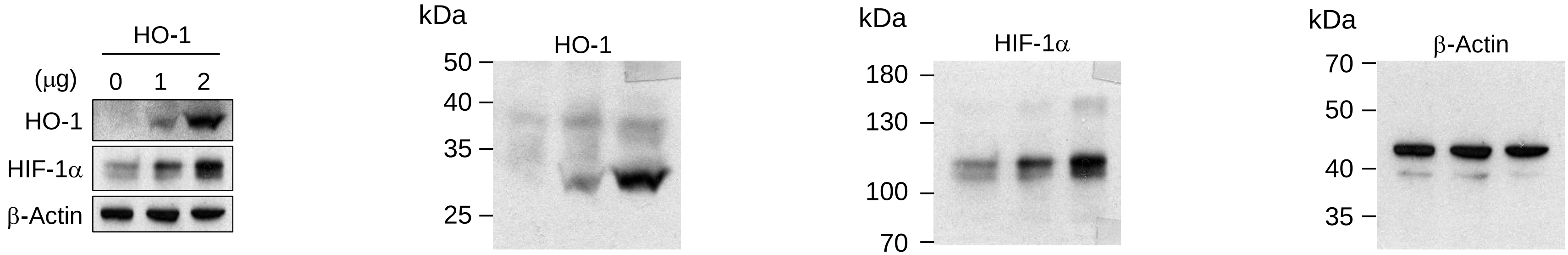

Fig 2E

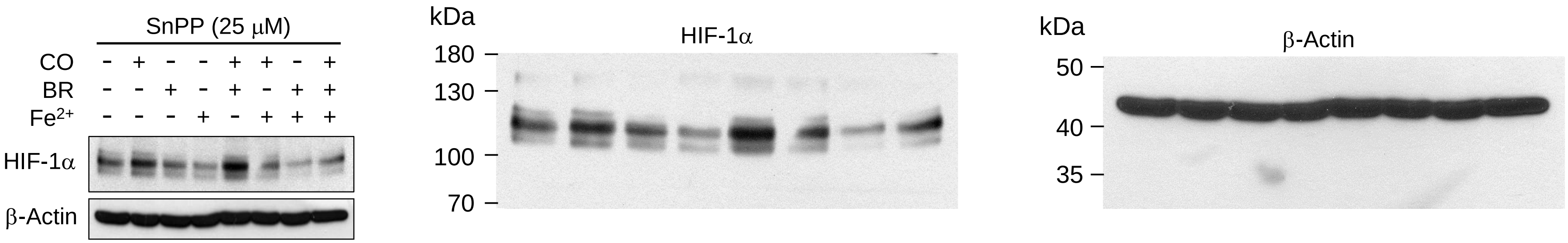

Fig 2F

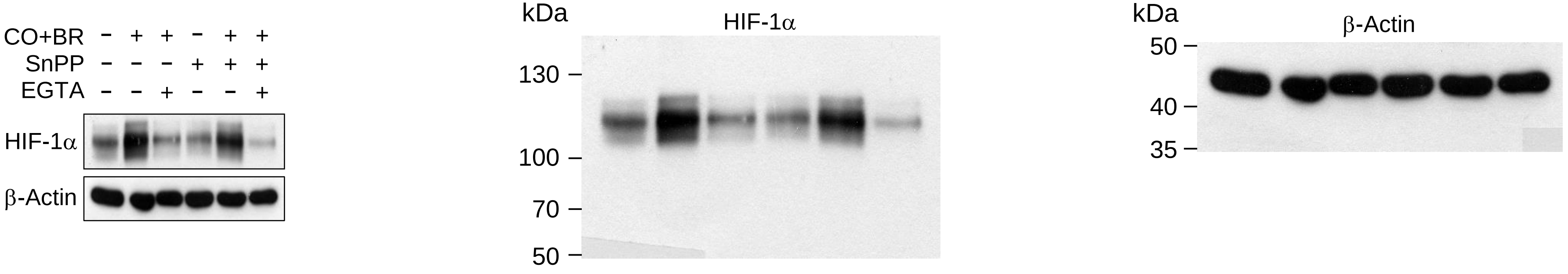

Fig 2G

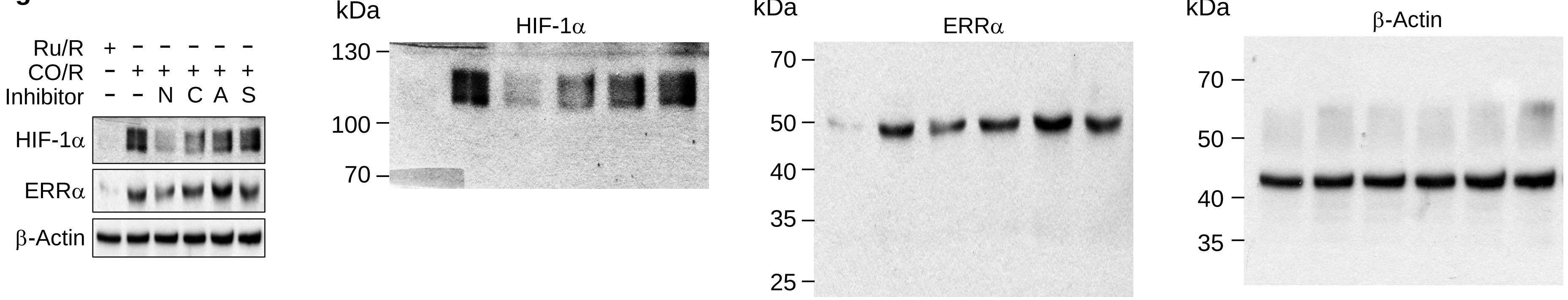

Fig 3A

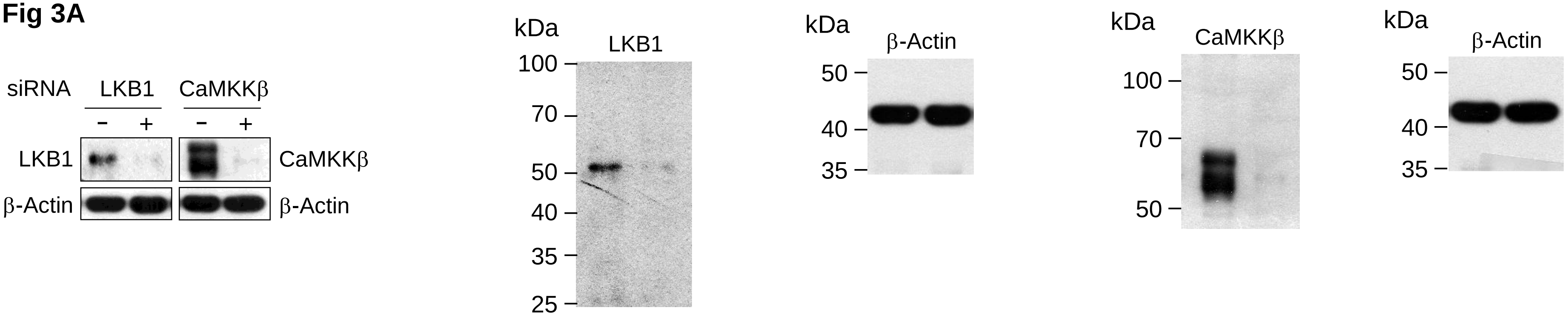

Fig 3B

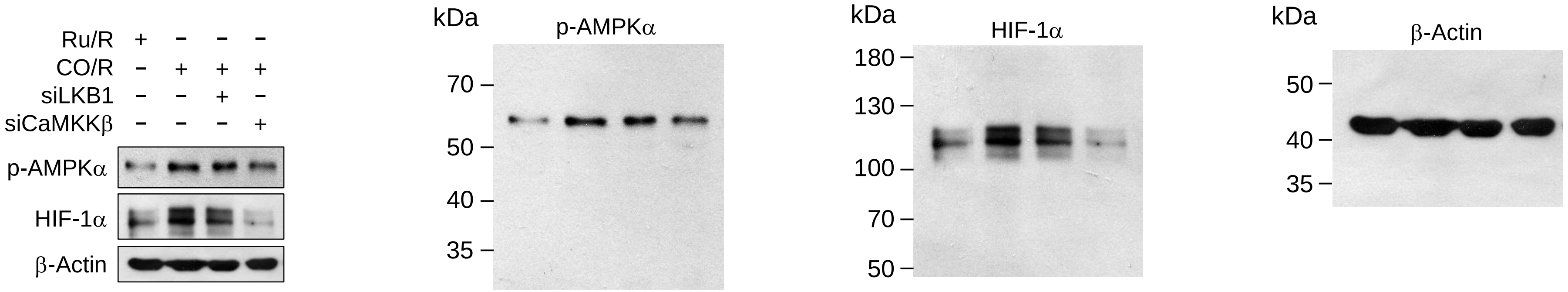
Ru/R
CO/R
siLKB1
siCaMKKβ
p-AMPKα
HIF-1α
β-Actin
kDa
70
50
40
35
180
130
100
70
50

Fig 3C

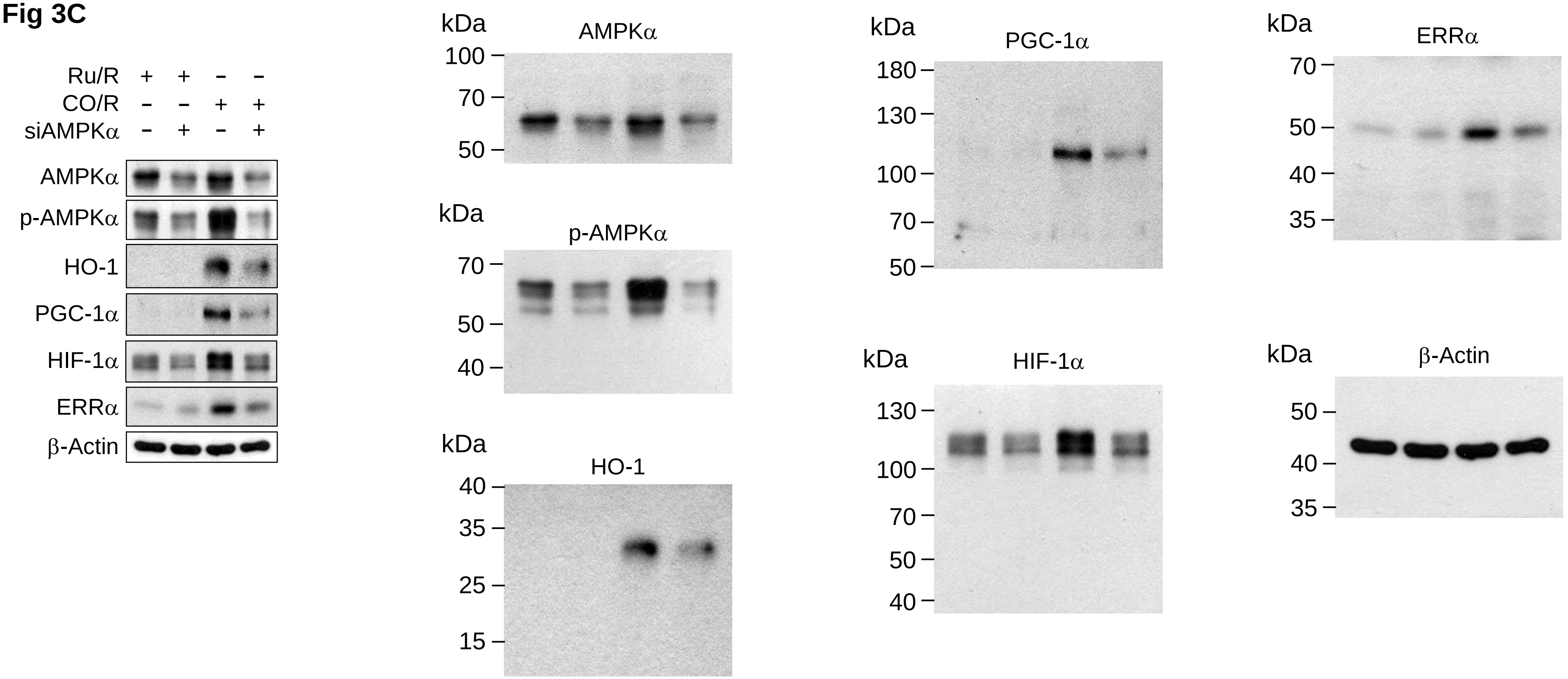
Ru/R
CO/R
siAMPKα
AMPKα
p-AMPKα
HO-1
PGC-1α
HIF-1α
ERRα
β-Actin
kDa
100
70
50
40
35
25
15
180
130

Fig 3D

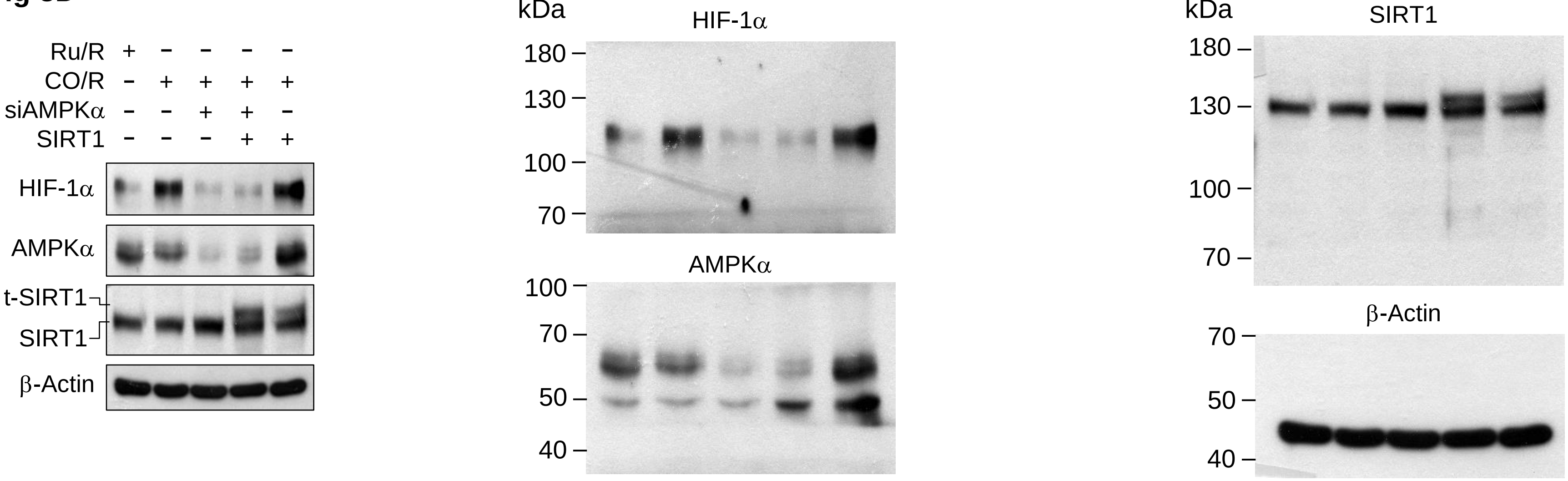
Ru/R + − − − −
CO/R − + + + +
siAMPKα − − + + −
SIRT1 − − − + +
HIF-1α
AMPKα
t-SIRT1
SIRT1
β-Actin
kDa
HIF-1α
180
130
100
70
AMPKα
100
70
50
40
kDa
SIRT1
180
130
100
70
β-Actin
70
50
40

Fig 4F

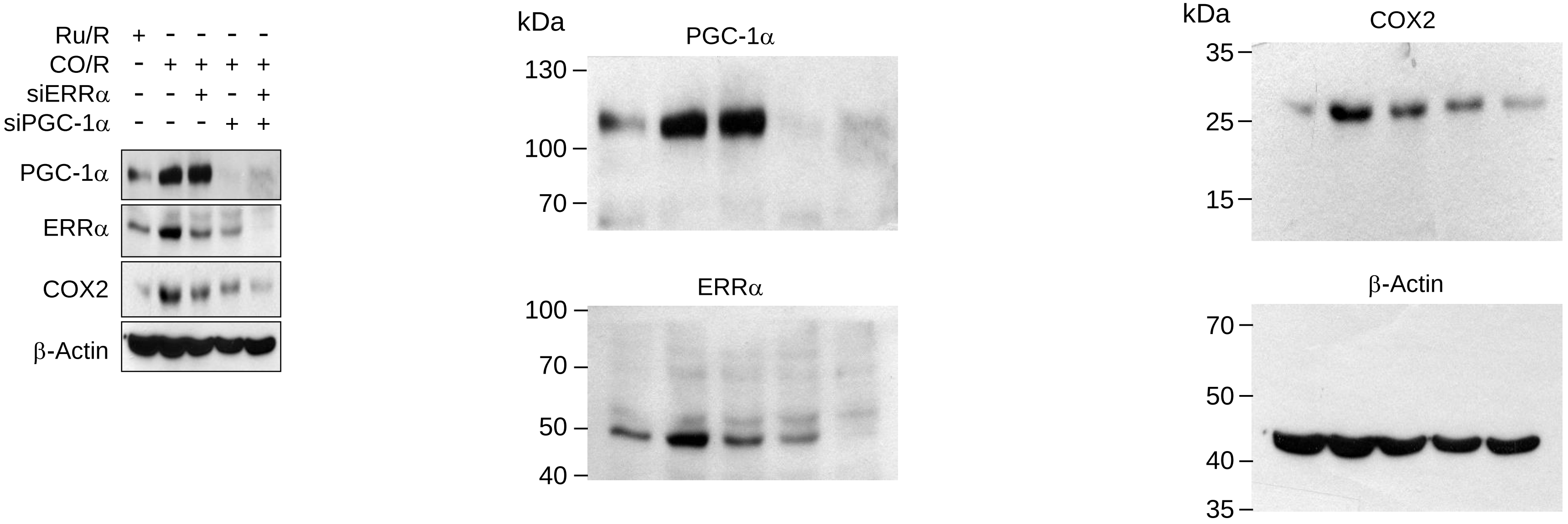
Ru/R + - - - -
CO/R - + + + +
siERRα - - + - +
siPGC-1α - - - + +
PGC-1α
ERRα
COX2
β-Actin
kDa
PGC-1α
130
100
70
ERRα
100
70
50
40
kDa
COX2
35
25
15
β-Actin
70
50
40
35

Fig 5A

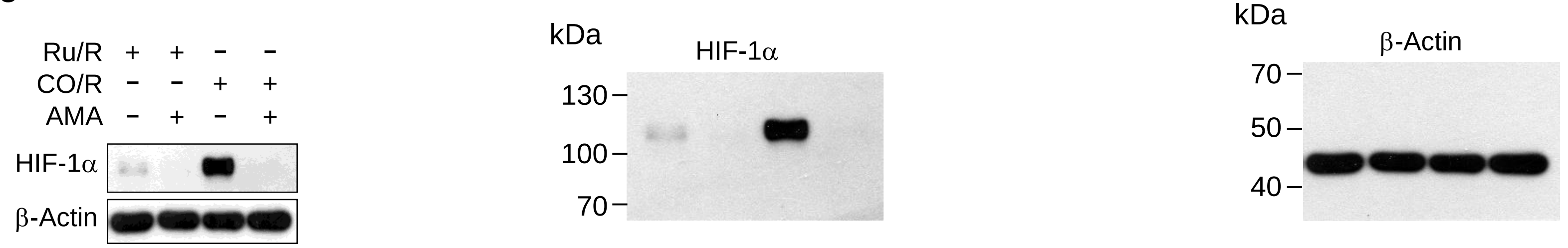

Fig 5B

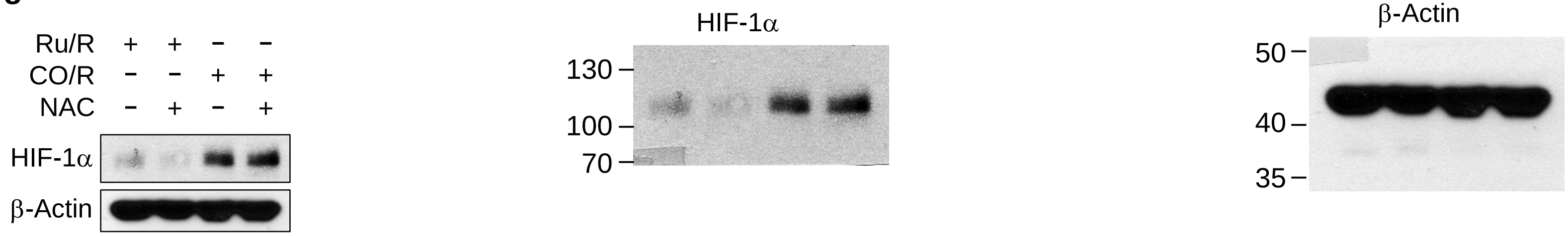

Fig 5C

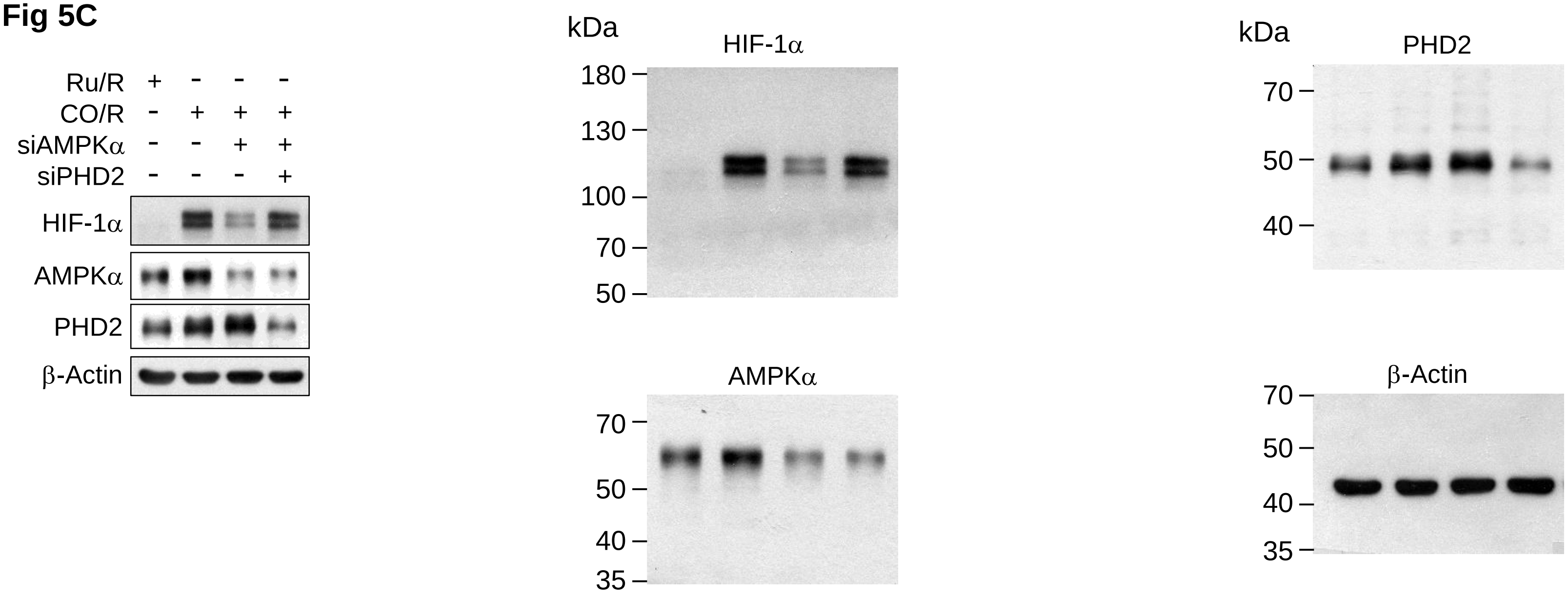

Fig 5D

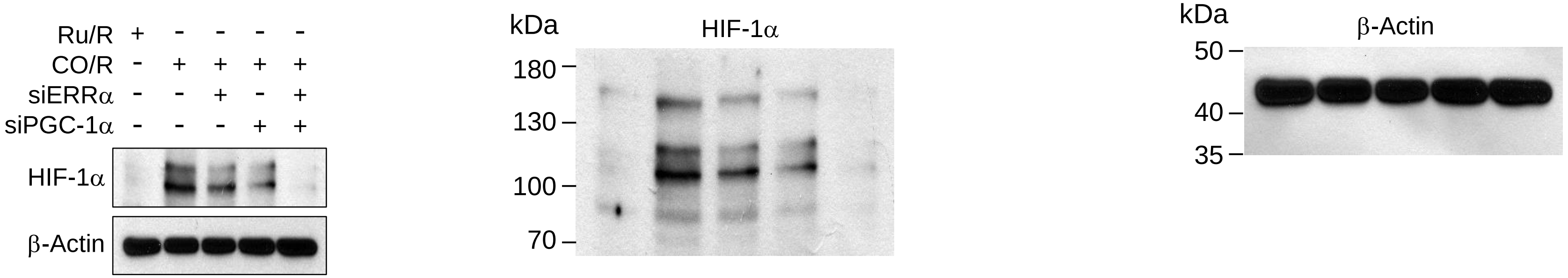

Fig 5F

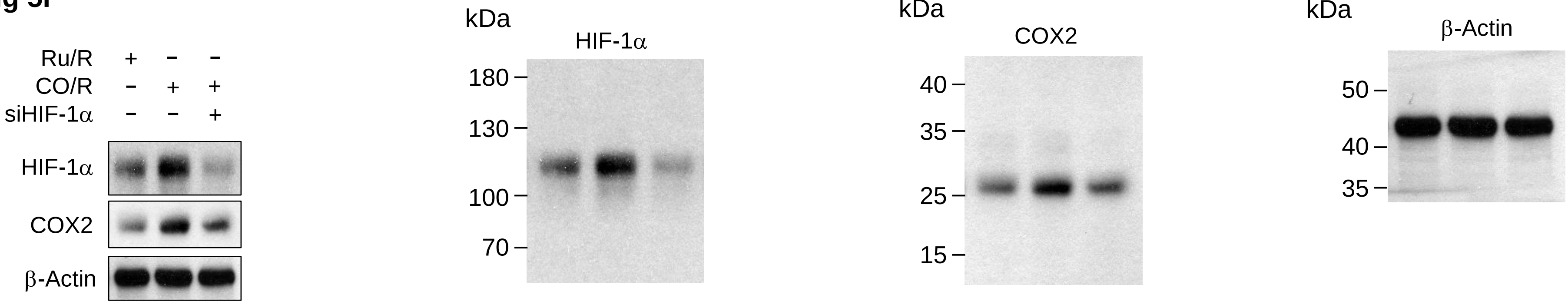

Fig 6A

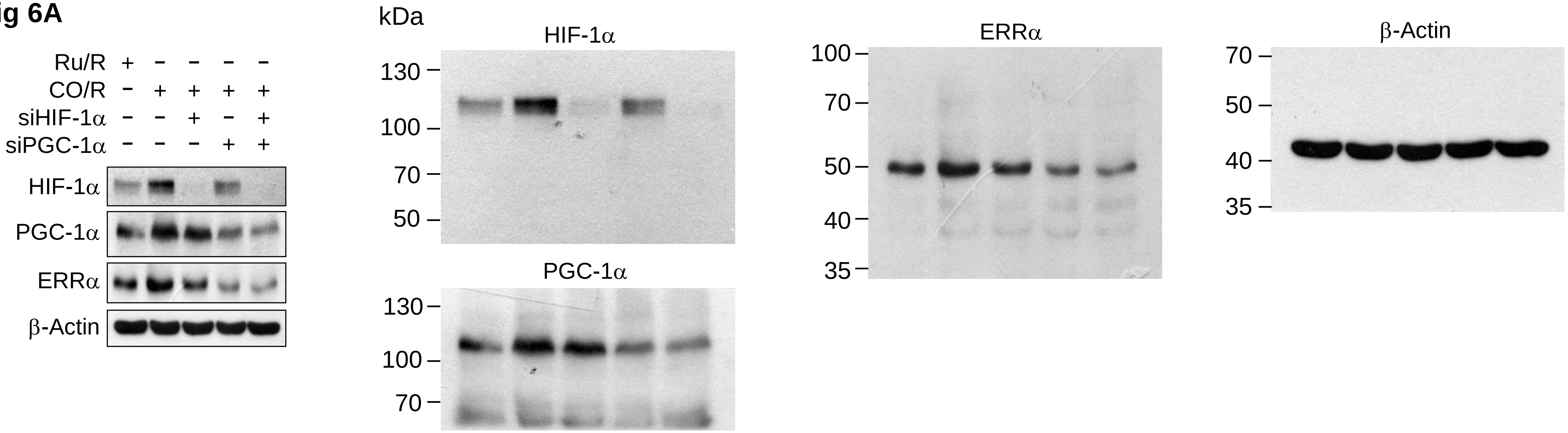

S1A Fig

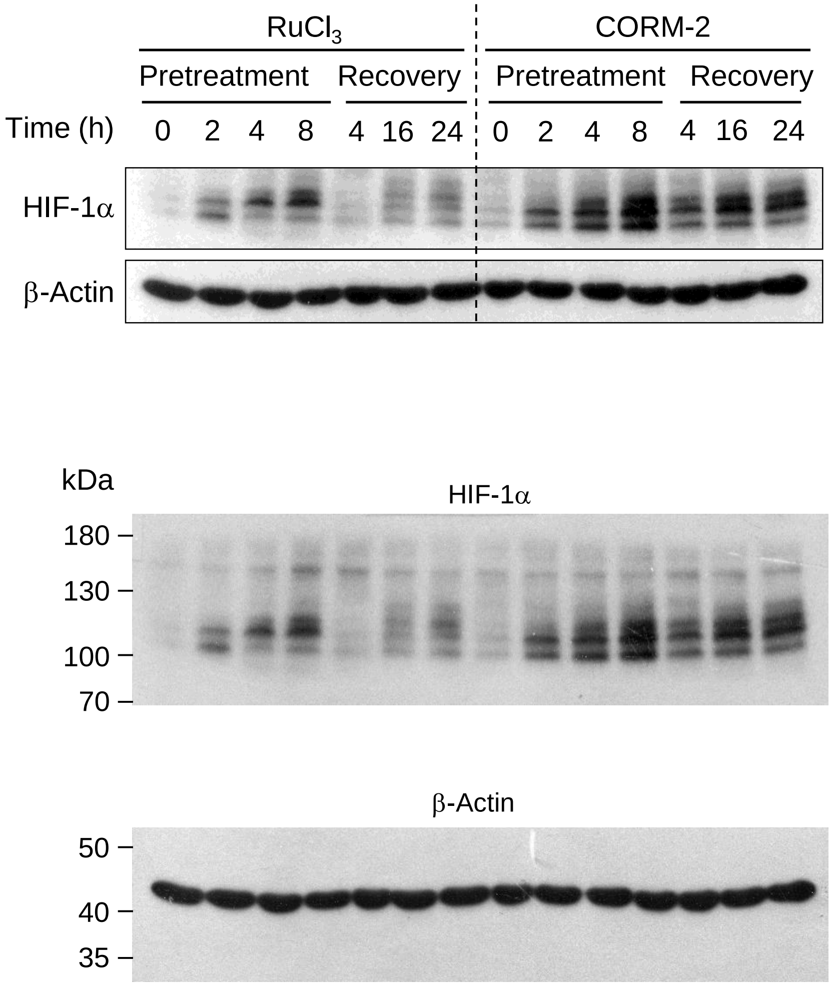

S1C Fig

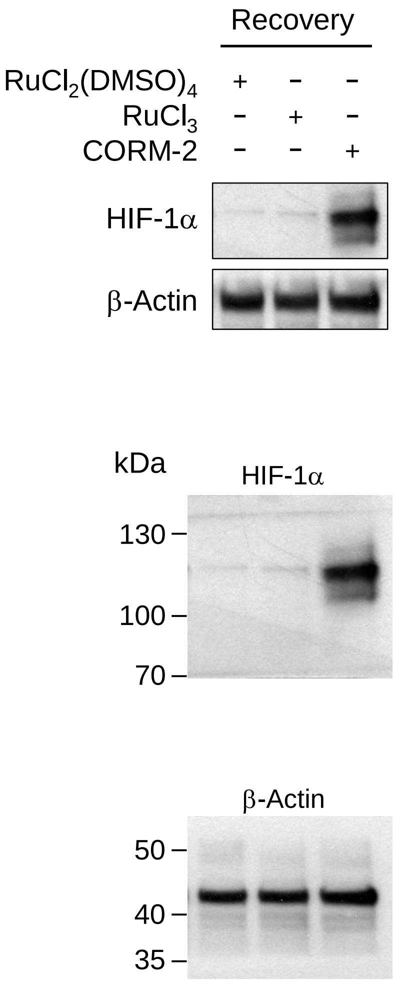

Supplement: S1 File — Membranes obtained from western blotting were demonstrated in this file. (PDF) [file pone.0202039.s002.pdf]
